# Synthesis of Selectively ^13^C/^2^H/^15^N‐ Labeled Arginine to Probe Protein Conformation and Interaction by NMR Spectroscopy

**DOI:** 10.1002/chem.202500408

**Published:** 2025-04-03

**Authors:** Darja I. Rohden, Giorgia Toscano, Paul Schanda, Roman J. Lichtenecker

**Affiliations:** ^1^ Faculty of Chemistry Institute of Organic Chemistry University of Vienna Währinger Str. 38 Vienna 1090 Austria; ^2^ Institute of Science and Technology Austria Am Campus 1 Klosterneuburg 3400 Austria; ^3^ Vienna Doctoral School in Chemistry (DoSChem) University of Vienna Währinger Str. 42 Vienna 1090 Austria; ^4^ MAG‐LAB Karl‐Farkas Gasse 22 Vienna 1030 Austria

**Keywords:** arginine labeling, protein interaction studies, protein NMR spectroscopy, stable isotope labeling

## Abstract

The charged arginine side chain is unique in determining many innate properties of proteins, contributing to stability and interaction surfaces, and directing allosteric regulation and enzymatic catalysis. NMR experiments can be used to reveal these processes at the molecular level, but it often requires selective insertion of carbon‐13, nitrogen‐15, and deuterium at defined atomic positions. We introduce a method to endow arginine residues with defined isotope patterns, combining synthetic organic chemistry and cell‐based protein overexpression. The resulting proteins feature NMR active spin systems with optimized relaxation pathways leading to simplified NMR spectra with a sensitive response to changes in the chemical environment of the nuclei observed.

## Graphical abstract

We offer a new labeling approach to introduce isolated ^15^N−^13^C−^1^H spin systems to the side chains of arginine residues. The labeling scheme delivers high‐resolution NMR spectra to study large protein complexes. The interaction between ubiquitin and the third Src homology 3 domain of yeast protein Sla1 (Sla1 SH3‐3) has been analyzed, highlighting the probes’ competence to sample rapid dynamics.

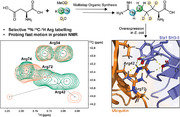

## 1 Introduction

The dynamic exchange of conformational states is crucial to protein function.^[^ ^1^, ^2^ ^]^ Modern NMR spectroscopy provides an extensive array of techniques to analyze protein dynamics, interactions, and structure of proteins in solution.^[^ ^3^, ^4^, ^5^, ^6^, ^7^, ^8^, ^9^ ^]^ The majority of these experiments require the insertion of NMR active spin 1/2 nuclei like carbon‐13 and nitrogen‐15 or/and the replacement of protons with deuterons to reduce spectral complexity, increase resolution, and optimize magnetization transfer pathways.^[^ ^10^, ^11^, ^12^, ^13^ ^]^ While many experiments exclusively focus on the protein backbone, it becomes more and more evident that the full potential of protein NMR can only be released when selective isotope patterns, which are tailored to pulse sequences aiming for side chain detection, are considered.^[^ ^14^, ^15^, ^16^, ^17^, ^18^, ^19^ ^]^ Especially, the functional groups at the end of longer side chains display additional conformational dynamics, which are largely independent from backbone motion. The positively charged arginine side chain is an important interaction partner in the binding to other proteins, nucleic acids, or small molecules.^[^ ^20^, ^21^, ^22^, ^23^, ^24^ ^]^ The guanidinium moiety of arginine contributes to cation–π and π–π interactions, contains up to five hydrogen bond donors, and is frequently involved in stabilizing salt bridges.^[^ ^25^, ^26^ ^]^ Unlike other positively charged residues, such as lysine or histidine, arginine retains its ionized state even in the hydrophobic core of folded proteins under all biologically relevant pH values due to the stabilizing mesomeric effect of the guanidinium group.^[^ ^27^, ^28^ ^]^ Fast solvent‐hydrogen exchange and the resulting line broadening often impedes the application of routine ^1^H—^15^N HSQC spectra when probing the arginine side chain.^[^ ^20^, ^21^, ^29^ ^]^ Unlike in the case of many aliphatic (Ile, Leu, Val)^[^ ^18^, ^19^ ^]^ or aromatic residues (Phe, Tyr, Trp),^[^ ^17^, ^30^, ^31^ ^]^ the bacterial biosynthesis of arginine features no direct α‐ketoacid precursor.^[^ ^32^ ^]^ Therefore, adding a more simple, achiral labeled compound from the biosynthetic pathway to the medium of a bacterial overexpression system for selective labeling of the target amino acid is not possible.

**Figure 1:**
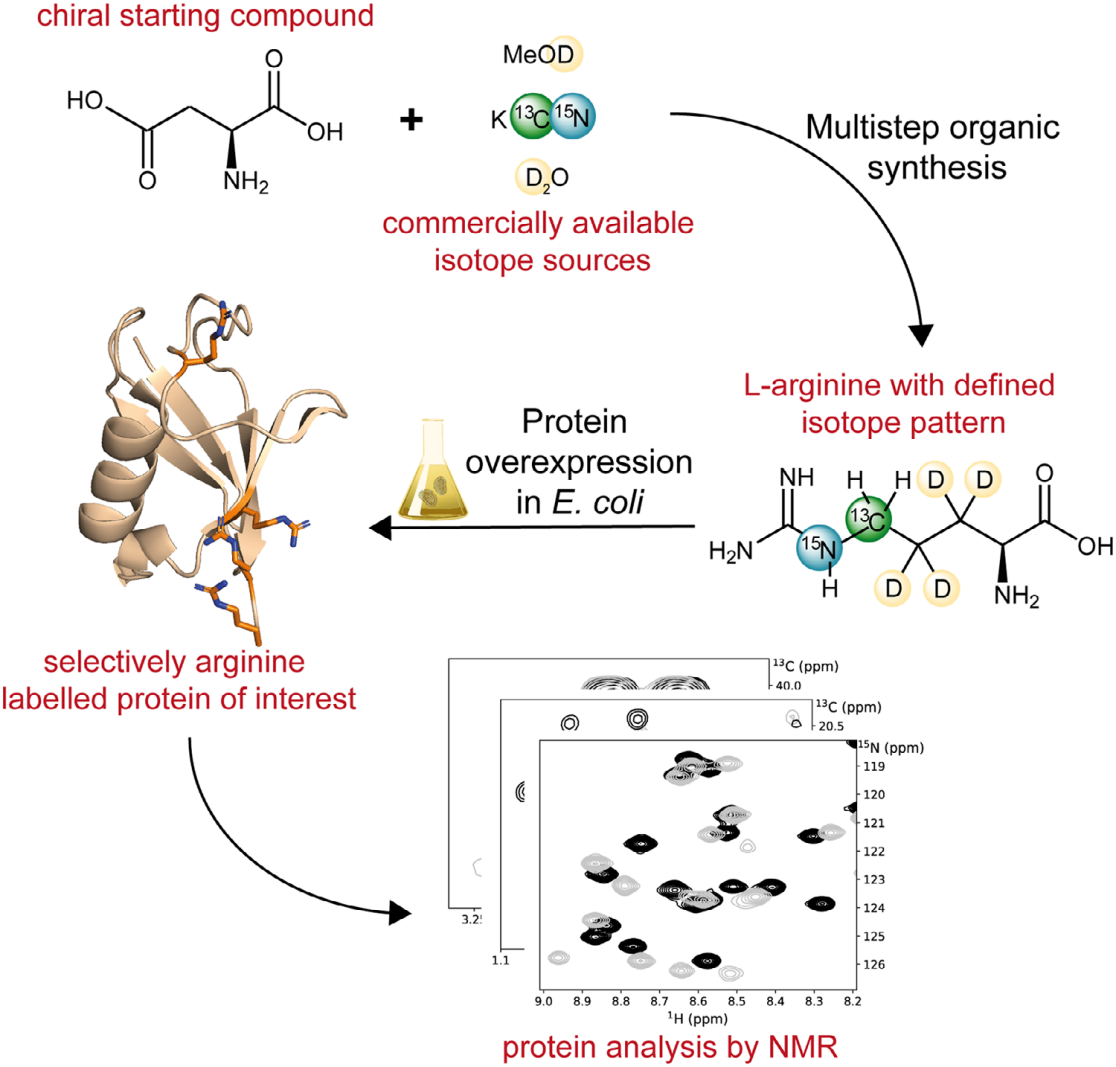
General overview of the approach developed. Commercially available non‐expensive sources of heavy isotopes are used in a multistep organic synthesis route to access selectively labeled l‐arginine. This compound is applied in protein overexpression techniques to endow target proteins with defined isotope patterns in arginine side chains. The resulting ^13^C─^1^H or ^13^C─^15^N resonances are ideal sensors for protein NMR applications.

Figure 1 summarizes our approach presented in this article: we implemented a synthetic route to access ^13^C_δ_/^2^H_βγ_/^15^N_ε_ arginine **12**. To investigate the incorporation efficiency, we used this isotope‐ labeled Arg in the *Escherichia coli*–based overexpression of the model protein ubiquitin. Moreover, we showed the utility of our approach by studying the interaction of ubiquitin and the third Src homology 3 domain (SH3) of the cytoskeletal protein binding domain Sla1.^[^ ^33^ ^]^ Our method is a general approach to explore, for example, dynamics and interaction of arginine side chains, taking advantage of well‐resolved ^1^H─^13^C or ^13^C─^15^N correlations in future studies. The full potential of our labeling pattern emerges when applied to a large deuterated protein samples, where arginine δ‐protons remain isolated in a fully deuterated environment.

## 2 Results and Discussion

### 2.1 Synthesis of ^13^C_δ_/^2^H_βγ_/^15^N_ε_ Arginine

**Scheme 1:**
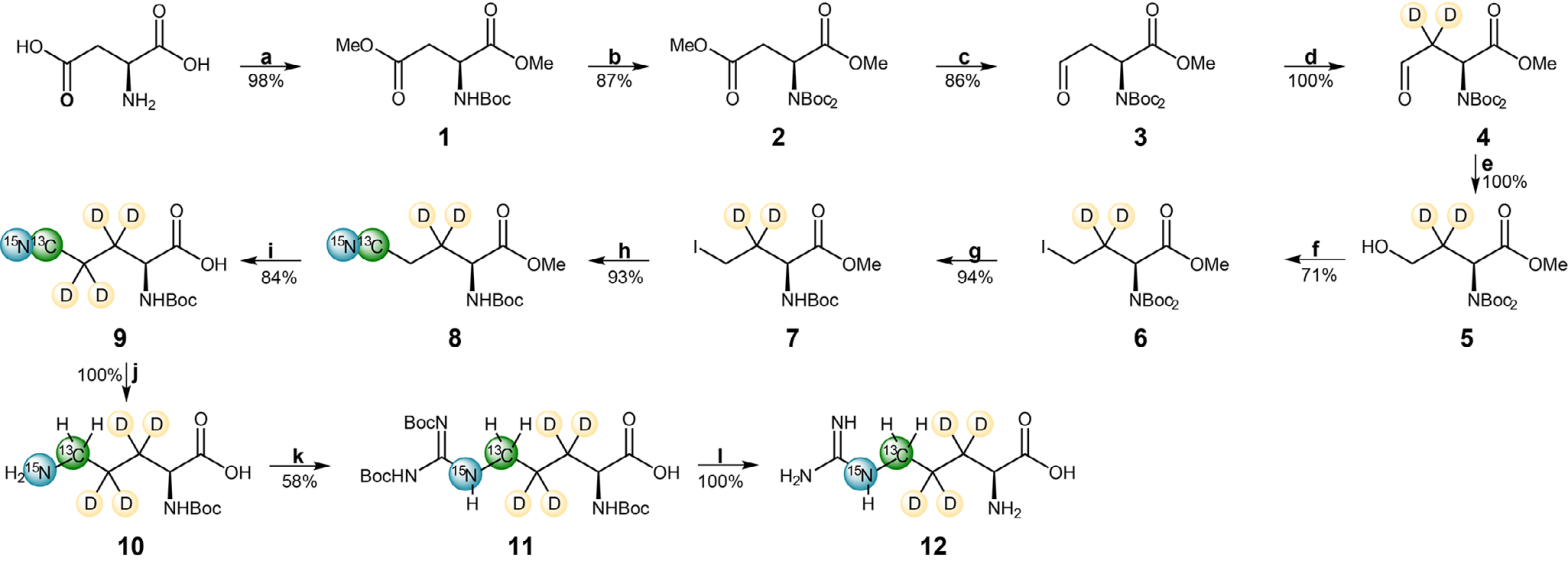
Synthesis route to yield ^13^C_δ_/^2^H_βγ_/^15^N_ε_ l‐arginine. a) 1. TMSCl, MeOH, 0 °C to RT, 16 hours; 2. (Boc)_2_O, Et_3_N, 0 °C to RT, 16 hours; b) (Boc)_2_O, DMAP (cat.), MeCN, RT, 16 hours; c) DIBAL‐H, Et_2_O, −78 °C, 3 hours; d) MeOD, Et_3_N (cat.), RT, 65 hours; e) NaBH_4_, MeOH, −10 °C, 15 minutes; f) PPh_3_, I_2_, Imidazole, THF, RT, 50 minutes; g) TFA, CH_2_Cl_2_, −10 °C, 1 hour; h) K^13^C^15^N, DMF, RT, 16 hours; i) DBU, D_2_O, 100 °C, MW, 1 hour; j) Pd/c, H_2_, MeOH, RT, 16 hours; k) N,N‐bis‐Boc‐1‐guanyl pyrazole, MeOH, 50 °C, 1 hour; l) TFA, CH_2_Cl_2_, RT, 16 hours.

The synthetic route to access the target arginine isotope pattern (**12** in Scheme 1) was designed based on the following considerations: 1) The use of low‐cost isotope sources. 2) The generation of a final product with highest possible enantiopurity, either by using a chiral starting compound or applying a stereocontrolled reaction step. 3) ^13^C incorporation as late as possible in the reaction sequence. 4) A reaction route with certain flexibility to allow for the synthesis of different isotopologues. The corresponding synthetic route is shown in Scheme 1. We chose a literature‐reported procedure describing the synthesis of 5–^13^C arginine as a starting point,^[^ ^34^ ^]^ which we modified and extended to the introduction of ^15^N. To diminish line‐broadening resulting from ^1^H–^1^H dipolar interactions, we implemented deuterium in the positions β and γ.

Commercially available l‐aspartate was converted into the corresponding *N*‐Boc‐protected dimethyl ester **2** in two steps in 85% yield. Introducing two Boc‐protecting groups, enabled for the reduction of the *β*‐methyl ester in a regioselective manner using diisobutylaluminium hydride (DIBAL‐H) as reported previously in the literature.^[^ ^34^, ^35^, ^36^ ^]^ The slow, portion‐wise addition of the reducing agent was crucial in this step to avoid over‐reduction to the corresponding alcohol side‐products (see Experimental Section for details). Aldehyde **3** was quantitatively deuterated under basic conditions in MeOD before reducing the carbonyl to the primary alcohol (**4**→**5**). A subsequent Appel iodination gave intermediate **6** in 71% yield. The literature procedures suggest the nucleophilic introduction of the cyano group prior to the partial deprotection of the amine.^[^ ^34^ ^]^ However, due to the possibility of an undesired Ritter‐type side reaction of the nitrile with a *tert*‐butyl cation, which is released, we decided to first remove the Boc‐protection group by subjecting intermediate **6** to trifluoroacetic acid (TFA) yielding compound **7** almost quantitatively. The stable isotopes ^13^C and ^15^N were introduced by displacing the iodine using [^13^C^15^N] potassium cyanide to give nitrile **8** in 93% yield.

We chose not to introduce the ^2^H at γ‐positions during the steps (c) and (e) using deuterated reducing agents, due to the high costs of DIBAL‐D and NaBD_4_. It is more cost effective to introduce deuterium at a later stage. Therefore, we focused on exchanging the protons alpha to the nitrile for deuterium while hydrolyzing the methyl ester to the free acid using deuterium oxide as a solvent. While in protocols published previously, this hydrolysis was performed under alkaline conditions using 1.5 equivalents of lithium hydroxide,^[^ ^34^ ^]^ these procedures did not lead to simultaneous deuteration. Increasing temperature or concentration of base led to nitrile hydrolysis giving Boc‐protected glutamic acid. Therefore, we screened reaction conditions based on the method of Luo and co‐workers, ^[^ ^37^ ^]^ who reported a deuteration strategy for nitriles in D_2_O at room temperature using trimethylamine as base. The method was applied and optimized as indicated in the Supporting Information (Table S1), which led to ester hydrolysis even without addition of lithium compounds, but failed to result in any deuteration at the desired position. In further reaction optimization, triethylamine was exchanged to the stronger, nonnucleophilic base diazabicycloundecene (DBU). To improve deuteration, the reaction was conducted under microwave irradiation at a higher temperature. A deuteration grade of > 90% was achieved with a substrate concentration of 0.13 M at 100 °C. Longer reaction times resulted in undesired nitrile hydrolysis. The deuterated nitrile **9** was subjected to hydrogenation in the presence of palladium on carbon. This step gave the free amine **10** quantitatively, which was treated with *N,N*‐bis‐Boc‐1‐guanyl pyrazole to give the tri‐Boc protected arginine analogue **11** in 58% yield. Final deprotection using TFA resulted in ^13^C_δ_/^2^H_βγ_/^15^N_ε_ l‐arginine **12** with an overall yield of 22% over 12 steps. The isotope source K^13^C^15^N was introduced in step 8 with two equivalents and was retained with a yield of 23%, which corresponds to 160 mg of K^13^C^15^N needed for 100 mg of final compound. We calculated the average material costs to produce arginine isotopologue **12** to €385 per 100 mg. Thus, our method not only improves linewidths due to deuteration of the β and γ position, but is also more cost‐effective than uniformly ^13^C (and ^15^N) labeled arginine from commercial vendors, which often exceeds €1.400 per 100 mg.

### 2.2 Incorporation into a Model Protein

**Figure 2:**
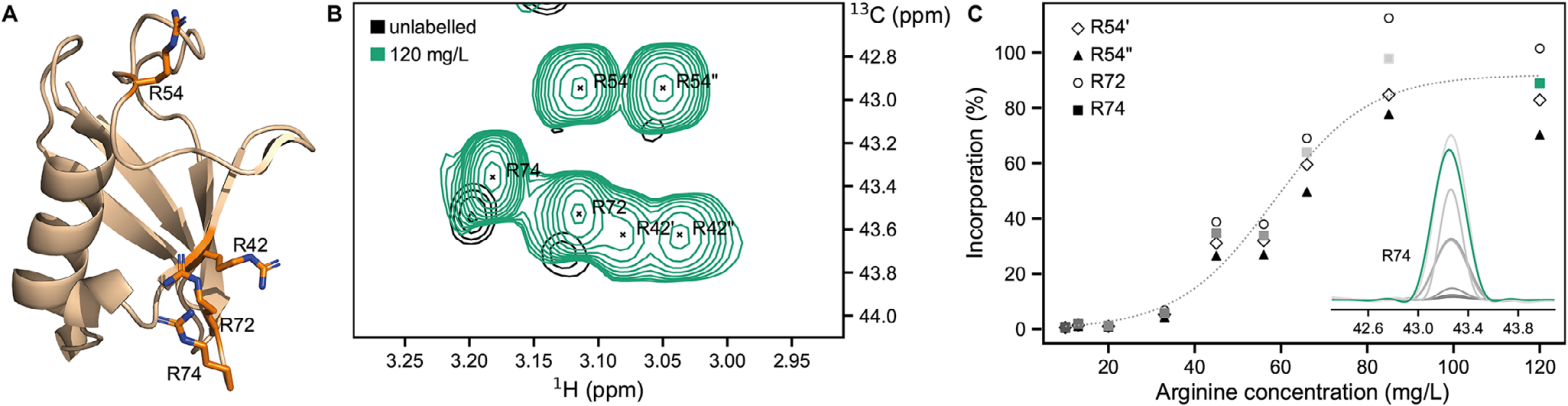
Incorporation study for residue‐specific labeling of ubiquitin with ^13^C_δ_/^2^H_βγ_/^15^N_ε_ l‐arginine. **A** Structure of ubiquitin (PDB: 4XOL). Arginines are shown in orange with a ball‐and‐stick representation. **B** ^1^H─^13^C HSQC showing the labeled ^13^C_δ_/^1^H_δ_ signals (green) in comparison to an unlabeled sample (black). Here, the spectra were normalized to the methyl region (see Experimental Section), such that intensities can be compared (see panel **C**). Note that the natural‐abundance signals of R42‐^13^C_δ_/^1^H_δ_ are of low intensity and close to the noise, despite a concentration of 500 µM and a measuring time of 1.5 hours at an 800 MHz spectrometer. These signals were not considered to calculate incorporation rate. **C** Labeled arginine concentration versus incorporation rate for individual arginine signals. A dotted line represents sigmoidal fit of mean values for each concentration. 1D spectrum in the right corner shows slices extracted at 3.18 ppm from spectra acquired from samples with different arginine concentrations.

We used human ubiquitin as a model protein to test the application of compound **12** for selective arginine labeling. The 76 amino acid–long protein is highly conserved among the animal and plant kingdoms and has several crucial biological roles. The most studied is its covalent linkage to label proteins for proteolytic degradation.^[^ ^38^ ^]^ The structure of ubiquitin is well studied and both NMR as well as X‐ray crystallographic data are available.^[^ ^39^, ^40^ ^]^ The secondary structure (Figure 2A) consists of five *β*‐strands, one *α*‐helix, a long loop region connecting strand four and five, and a highly flexible C‐terminus, which is known to interact with diverse ubiquitin binding proteins (UBPs).^[^ ^41^ ^]^ We produced a uniformly ^15^N‐ labeled ubiquitin sample in an *E. coli* overexpression system containing ^13^C_δ_/^2^H_βγ_/^15^N_ε_ l‐arginine **12,** which was added 30 minutes prior to induction, to ensure sufficient uptake of the compound. To screen optimal incorporation parameters, we varied the concentration of labeled amino acid from 10 to 120 mg/L and analyzed the isotope incorporation using ^1^H–^13^C NMR spectra (Figure 2B). We calculated the incorporation rate by comparing the peak heights of arginine‐^13^C_δ_/^1^H_δ_ of labeled samples to those of unlabeled ubiquitin (see Experimental Section for more details). The resulting graph (Figure 2C) shows that at concentrations of 80–120 mg/L more than 85% isotope incorporation can be achieved. We observe a sigmoidal curve with a very flat initial slope, i.e., low arginine incorporation up to a concentration of 40 mg*/*L, which suggests that the arginine added to the culture is used in other metabolic pathways. Arginine is part of the urea cycle and an important precursor in the polyamine pathway.^[^ ^42^ ^]^ Further, an amidinotransferase can transfer the guanidino group to glycine yielding guanidinoacetate and ornithine.^[^ ^43^ ^]^ While the latter is used as a nitrogen source within the creatine pathway, ornithine can be decarboxylated to give putrescine, which again constitutes an entry point to the polyamine pathway.^[^ ^44^ ^]^ Thus, we assumed that the addition of polyamines or other metabolites to the overexpression media might lead to an increased incorporation rate at low arginine concentrations. We produced 15 samples with additions of spermidine, putrescine, ornithine, guanidinoacetate, or GABA. These experiments suggest incorporation gains upon addition of spermidine and ornithine (see Supporting Information, Figure S1): in all these experiments, the incorporation rate was at least as high as in the experiments without addition, and in several experiments, it was significantly higher. However, further investigation is needed to quantify the effects, which is beyond the scope of this study.

The ^1^H─^13^C HSQC spectrum of ^13^C_δ_/^2^H_βγ_/^15^N_ε_ arginine‐labeled ubiquitin revealed very intense signals for ^13^C_δ_/^1^H_δ_ correlations (Figure 2B). Due to the isotope effect of the neighboring deuterons, ^[^ ^45^, ^46^ ^]^ the signals in the labeled sample are shifted by ‐0.01 and ‐0.20 ppm in the ^1^H and ^13^C dimension, respectively. Two of the four arginines (R42 and R54) display two distinct frequencies in the proton dimension, reflecting the two diastereotopic δ‐protons. In contrast, for both R72 and R74 we observed a single ^1^H frequency, indicating rapid (sub‐millisecond) dynamics that averages the frequencies of the two diastereotopic δ‐protons. Both residues are located in the highly flexible C‐terminus of the protein.

**Figure 3:**
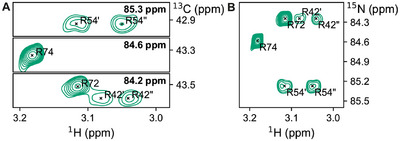
NMR spectra showing the signals for ^13^C_δ_/^2^H_βγ_/^15^N_ε_ l‐arginine in ubiquitin. **A** 2D planes obtained from a 3D H–C–N out‐and‐back experiment showing the labeled ^13^C_δ_/^1^H_δ_ signals at the different ^15^N frequencies. **B** 2D H–(c)–N version of the same experiment conducted in A.

We furthermore used the ^15^N nucleus in the ε position for experiments that correlate the δ‐protons, the δ‐carbon, and the ε‐nitrogen in either a 3D H–C–N out‐and‐back experiment (Figure 3A) or a 2D H–(c)–N version thereof (Figure 3B). The additional nitrogen dimension provides an increase in resolution, which, although not needed in ubiquitin, may prove useful in larger molecules containing more arginines. Due to its position in the charged guanidinium head group, the N_ε_ is also known to form salt bridges to nearby carboxylate groups, which introduces a displacement of its chemical shift by ca. 0.5 ppm.^[^ ^47^ ^]^ The developed arginine isotopologue can prove useful in probing these interactions in a more precise manner.

### 2.3 Arginines Probe the Binding of Ubiquitin to Sla1 SH3‐3

**Figure 4:**
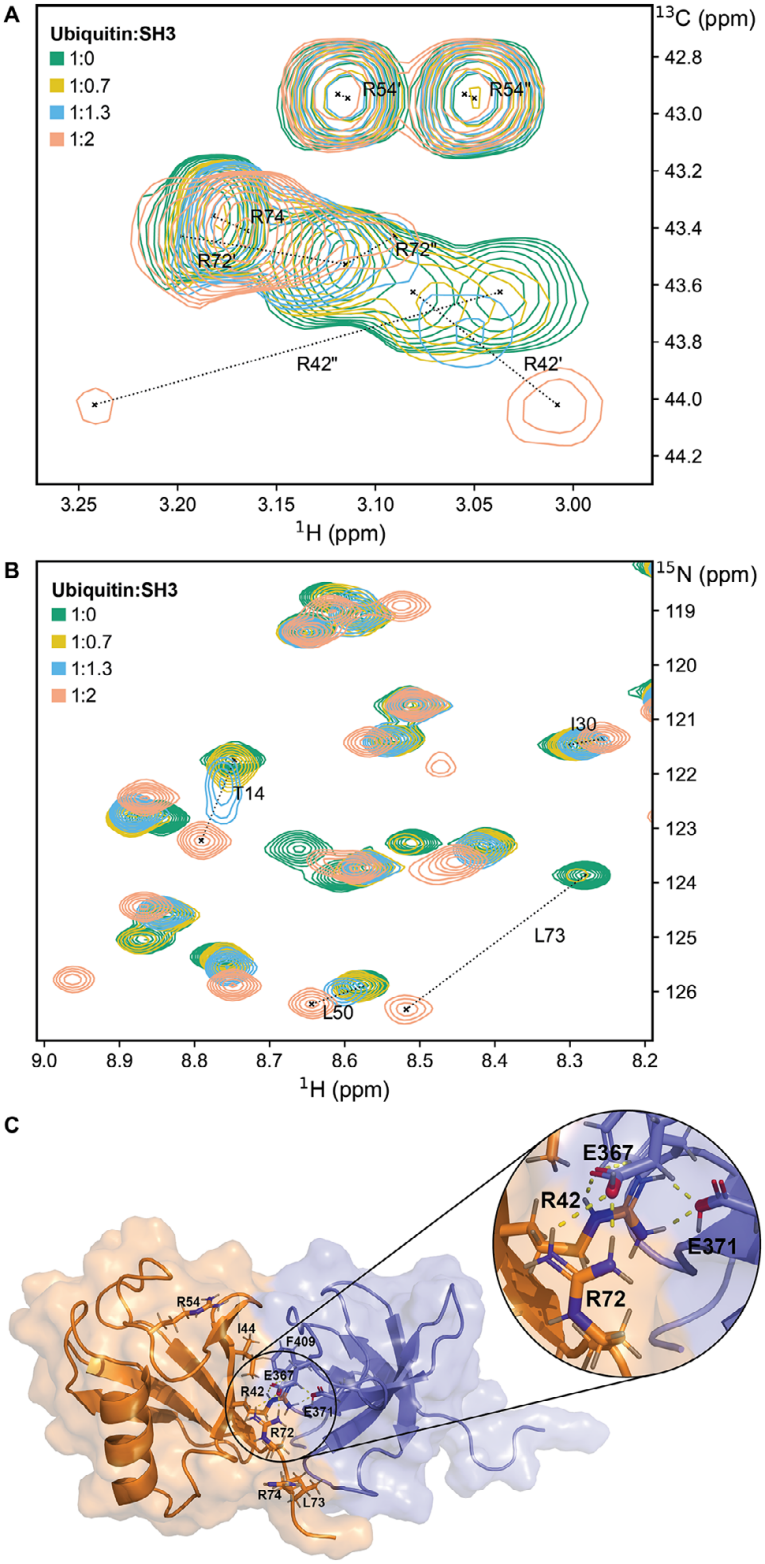
NMR spectra of U‐^15^N, ^13^C_δ_/^2^H_β__γ_/^15^N_ε_ arginine‐labeled ubiquitin titrated with Sla1 SH3‐3. Proteins were dissolved in 50 mM Tris, pH 8.0. **A** ^1^H–^13^C HSQC showing the labeled ^13^C_δ_/^1^H_δ_ signals. **B** ^1^H–^15^N SOFAST‐HMQC showing backbone NH signals. **C** One conformer of the NMR structure ensemble showing the complex between ubiquitin (orange) and Sla1 SH3‐3 (blue) according to PDB 2JT4. The side chains of arginines, L73, and I44 (ubiquitin), as well as E367, E371, and F409 (SH3) are indicated by a ball‐and‐stick representation.

Having optimized the synthetic route to the arginine isotopologue **12** and its application in bacterial overexpression of proteins, we demonstrated the usefulness of this specific labeling for monitoring protein–protein interactions, using the Src homology 3 (SH3) domain, a previously characterized ubiquitin binder.^[^ ^33^, ^48^, ^49^ ^]^ We chose the third SH3 domain of the yeast protein Sla1 (Sla1 SH3‐3) as the interaction partner for our labeled ubiquitin sample. Sla1 acts as a regulatory adaptor protein, linking the site of endocytosis to the actin cytoskeleton, and its SH3‐3 domain has been shown to selectively bind ubiquitin with a *K*_d_ of ∼40 µM.^[^ ^33^, ^50^ ^]^ NMR‐based structural models for the ubiquitin complex interface with Sla1 SH3‐3, as well as its mammalian orthologue CIN85‐SH3‐C are available.^[^ ^48^, ^51^ ^]^ The binding site of ubiquitin and Sla1 SH3‐3 has been identified.^[^ ^33^, ^52^, ^53^ ^]^ It involves the C‐terminal end of the β‐strand 5, also known as the hydrophobic patch (comprising R42), and the adjacent C‐ terminus (comprising R72 and R74, Figure 4). Arginines are ideally positioned to probe this interaction.

We expressed the non labeled Sla1 SH3‐3 in a cell‐free expression system based on *E. coli* S15 extract and studied the chemical shift perturbations of arginine‐^13^C_δ_/^1^H_δ_ signals in arginine‐labeled ubiquitin upon binding to the SH3 domain (Figure 4A). As expected, the signals of R42 and R72 shift significantly while R54 stays mostly unaffected during the titration. The relatively small shift of 0.01 ppm for R74 leads to the conclusion that the side chain of this residue does not directly participate in binding. Interestingly, the arginine signals reveal that binding impacts the dynamics of the C‐terminus: while in the apo protein the R72 side chain undergoes sub‐millisecond dynamics that lead to a single averaged δ‐proton signal, two distinct signals are observed in the complex. This observation unequivocally shows that the side chain of R72 exists in a well‐defined position rather than as rapidly interconverting states. The available NMR models for the complex between Ubiquitin and Sla1 SH3‐3 further underline this observation^[^ ^48^ ^]^ (Figure 4C): R72, similar to R42, is positioned at the binding interface and thus can directly interact with glutamates at the surface of SH3. The observed chemical shift perturbations and the signal splitting proves the contribution of R72 to the overall binding with the Sla1 SH3‐3 domain. The signals of R42 experience a shift of 0.12 and 0.23 ppm and significantly broaden in the course of the titration, which is indicative for intermediate exchange dynamics, possibly due to binding and release of the complex and/or dynamics within the complex. Similar behavior is also visible in the amide ^1^H–^15^N spectra (Figure 4B) and reproduces available literature data: The arginine‐neighboring L73 experiences a strong chemical shift perturbation and peak broadening, which further justifies this area being important for binding.^[^ ^33^ ^]^

## 3 Conclusion

We have developed a method for incorporating ^1^H_2_–^13^C_δ_ or ^1^H_2_–^13^C_δ_–^15^N_ε_ spins in otherwise deuterated ^12^C side chains of arginine. When overexpression is performed in a D_2_O‐based medium, our approach results in proteins that are fully deuterated except at the δ position of arginine. Unlike the case of methyl‐labeling strategies, the method involves the synthesis of the amino acid instead of a metabolic precursor, which expands its application beyond bacterial overexpression to the use in cell‐free protein synthesis or eukaryotic protein production.

Our approach does not result in stereoselective protonation of only one of the two δ hydrogen sites, and consequently two peaks are observed per arginine. In the presence of rapid dynamics that average the two environments of the δ hydrogens, the peaks collapse, thus providing a simple means of detecting fast motion. As illustrated, for the ubiquitin‐SH3 example, complex formation can lead to a rigidification of the side chain, which is readily detected by the splitting into two distinct peaks. While the current study has focused on the synthesis of the amino acid and its use in small proteins, we foresee that the combination of our approach with methylene‐TROSY^[^ ^54^ ^]^ will be particularly beneficial for larger proteins. A methylene‐TROSY‐based CPMG approach was developed for relaxation dispersion measurements in RNA,^[^ ^55^ ^]^ and it could be directly applied to samples generated herein. Since ^13^C–^13^C couplings can significantly interfere with such experiments,^[^ ^56^ ^]^ our labeling scheme of isolated ^13^CH_2_ groups offers the potential to serve as an effective source of accurate relaxation data in arginine‐containing proteins. We additionally expect the proposed approach to become useful for magic‐angle spinning (MAS) NMR applications. Deuteration and sparse protonation has allowed to dramatically improve the resolution of proton‐detected MAS NMR,^[^ ^57^ ^]^ whereby linewidths are independent of the size of the molecule. The fact that the Arg side chain contains a single ^13^C spin will also greatly facilitate the interpretation of spin relaxation experiments, both in solution and in solids. Collectively, the proposed method contributes to the arsenal of residue‐specific labeling methods. We believe that the ability to obtain high‐quality spectra of arginine will help decipher interactions and dynamics in the active sites of enzymes^[^ ^58^ ^]^ and binding interfaces,^[^ ^59^ ^]^ in which arginines are over‐represented and play an important role.

## 4 Experimental

**General procedures**: Unless otherwise stated, all reagents and reactants were purchased from commercial suppliers and used without further purification. Heptane and ethyl acetate were distilled prior to use, while all other solvents and reagents were used as received from commercial suppliers. Deuterated methanol was purchased from Eurisotop, [^13^C^15^N] potassium cyanide was purchased from Sigma Aldrich. Plasmids for protein expression were purchased from GeneCust. Oxygen‐ and moisture‐sensitive reactions were carried out under an argon atmosphere and yields refer to pure compounds. The reactions were monitored via thin‐layer chromatography (TLC) on silica gel 60 with fluorescent indicator UV254. Visualization of the compounds was carried out using an UV‐lamp (254 nm) and by application of ninhydrin staining solution with subsequent heating using a heat gun. Flash column chromatography was performed on silica gel 60 (0.040–0.063 nm) from Merck. High‐resolution mass spectrometry (HRMS) of organic small molecules was performed on Orbitrap Exploris 120 (Thermo Fisher Scientific) and on timsTOF fleX ESI/MALDI dual source–Qq‐TOF mass spectrometer (Bruker Daltonics) in the positive‐ and/or negative‐ion mode. The sum formulas of the detected ions were determined using Bruker Compass Data Analysis 5.3 based on the mass accuracy (∆*m*/*z* ≤ 5 ppm) and isotopic pattern matching (SmartFormula algorithm). Centrifugations were done in a refrigerated centrifuge Eppendorf 5910Ri, high‐speed centrifugations were carried out in a Thermo Scientific Sorvall LYNX 6000. *Escherichia coli* incubation was performed in an INFORS HT Multitron incubator at 37 °C with 200 rpm. Cell lysis was conducted using a Q700 Sonicator (QSonica).

### 4.1 Synthesis of ^13^C_δ_/^2^H_βγ_/^15^N_ε_ l‐arginine

**Dimethyl *N*‐(*tert*‐butoxycarbonyl)‐l‐aspartate (1)**: To a stirred solution of l‐aspartate (4 g, 30 mmol, 1 eq.) in dry methanol (60 mL), trimethylsilyl chloride (15.8 mL, 120 mmol, 4 eq.) was added slowly at 0 °C under inert conditions. The mixture was allowed to reach room temperature and stirred for 16 hours. Completion of esterification was indicated by clearance of the suspension into a colorless solution. The mixture was again cooled to 0 °C and triethylamine (TEA) (30 mL, 210 mmol, 7 eq.) was added slowly followed by introduction of di‐*tert*‐butyl dicarbonate (Boc_2_O) (10.6 mL, 45 mmol, 1.5 eq.). The reaction was proceeded another 16 hours at room temperature. After confirming full conversion of starting material by TLC (heptane/ethyl acetate 2:1), the reaction was quenched with 6 mL of H_2_O. The crude mixture was extracted three times with ethyl acetate and the combined organic layers washed with brine before drying over anhydrous MgSO_4_. The solvent was evaporated in vacuo and the residue purified by flash column chromatography (heptane/ethyl acetate 2:1). Product containing fractions were identified by ninhydrin staining and combined to give 7.68 g of title compound **1** as white solid (98%).

^1^H NMR (400 MHz, CDCl_3_) *δ*_H_: 5.48 (d, *J *= 7.6 Hz, 1H; NH), 4.58 (dt, *J =* 8.4, 4.3 Hz, 1H; C*_α_* H), 3.76 (s, 3H; OCH_3_), 3.69 (s, 3H; OCH_3_), 3.00 (dd, *J = *17.0, 4.3 Hz, 1H; C*_β_* H_2a_), 2.82 (dd, *J = *17.0, 4.7 Hz, 1H; C*_β_* H_2b_), 1.45 (s, 9H; Boc_CH3_). ^13^C NMR (101 MHz, CDCl_3_) *δ*_C_: 171.69 (C*_γ_* O), 171.58 (CO), 155.52 (Boc_CO_), 80.32 (Boc_tBu_), 52.87 (OCH_3_), 52.16 (OCH_3_), 50.06 (C*_α_*), 36.82 (C*_β_*), 28.44 (Boc_CH3_). HRMS: *m/z* C_11_H_19_O_6_N calc. for [M + Na]^+^ 284.1105; found 284.1115.

**Dimethyl *N,N*‐bis(*tert*‐butoxycarbonyl)‐l‐aspartate (2)**: To a solution of **1** (5.23 g, 20 mmol, 1 eq.) in 65 mL dry acetonitrile were added 4‐dimethylaminopyridine (DMAP) (489 mg, 4 mmol, 0.2 eq.) and Boc_2_O (7 mL, 30 mmol, 1.5 eq.) sequentially under inert conditions. The reaction was proceeded for 16 hours at room temperature and the conversion confirmed by TLC (heptane/ethyl acetate 3:1) before quenching with 13 mL of H_2_O. The crude mixture was extracted three times with ethyl acetate and the combined organic layers washed with brine solution before drying over anhydrous MgSO_4_. The solvent was evaporated in vacuo and the residue purified by flash column chromatography (heptane/ethyl acetate 4:1). Product‐containing fractions were identified by ninhydrin staining and combined to give 6.29 g of the title compound **2** as a white solid (87%).

^1^H NMR (700 MHz, CDCl_3_) *δ*_H_: 5.47 (t, *J = *6.8 Hz, 1H; C*_α_* H), 3.75 (s, 3H; OCH_3_), 3.73 (s, 3H; OCH_3_), 3.27 (dd, *J = *16.4, 7.1 Hz, 1H, C*_β_* H_2a_), 2.76 (dd, *J = *16.4, 6.5 Hz, 1H; C*_β_* H_2b_), 1.53 (s, 18H; Boc_CH3_). ^13^C NMR (176 MHz, CDCl_3_) *δ*_C_: 171.20 (C*_γ_* O), 170.44 (CO), 151.70 (Boc_CO_), 83.70 (Boc_tBu_), 55.05 (C*_α_*), 52.66 (OCH_3_), 52.10 (OCH_3_), 35.85 (C*_β_*), 28.11 (Boc_CH3_). HRMS: *m/z* C_16_H_27_O_8_N calc. for [M + Na]^+^ 384.1629; found 384.1633.

**(S)‐methyl‐2‐bis(*tert*‐butoxycarbonyl)amino‐4‐oxobutanoate (3)**: A solution of **2** (5.42 g, 15 mmol, 1 eq.) in 90 mL dry diethyl ether was cooled to − 78 °C under inert conditions. To this, a 1 M solution of DIBAL‐H in toluene (24 mL, 24 mmol, 1.6 eq.) was added slowly in four portions every 30 minutes. After the last addition, the reaction was proceeded another 90 minutes until complete conversion was observed by TLC (heptane/ethyl acetate 3:1). The reaction was removed from the cooling bath and quenched with 30 mL of H_2_O. After reaching room temperature, 200 mL of a saturated solution of potassium sodium tartrate was added and the mixture stirred vigorously for 16 hours. The phases were allowed to separate and the aqueous phase was extracted three times with ethyl acetate. The combined organic layers were washed with brine before drying over anhydrous MgSO_4_. The solvent was evaporated in vacuo and the residue purified by flash column chromatography (heptane/ethyl acetate 6:1). Product‐containing fractions were identified by ninhydrin staining and combined to give 4.27 g of the aldehyde **3** as a colorless oil (86%).

^1^H NMR (700 MHz, CDCl_3_) *δ*_H_: 9.79 (s, 1H; C*_γ_* HO), 5.53 (t, *J = *6.4 Hz, 1H; C*_α_* H), 3.73 (s, 3H; OCH_3_), 3.41 (dd, *J = *17.8, 6.8 Hz, 1H; C*_β_* H_2a_), 2.83 (dd, *J = *17.8, 5.9 Hz, 1H; C*_β_* H_2b_), 1.50 (s, 18H; Boc_CH3_). ^13^C NMR (176 MHz, CDCl_3_) *δ*_C_: 198.57 (C*_γ_* HO), 170.42 (CO), 151.84 (Boc_CO_), 83.87 (Boc_tBu_), 53.07 (OCH_3_), 52.77 (C*_α_*), 45.13 (C*_β_*), 28.12 (Boc_CH3_). HRMS: *m/z* C_15_H_25_O_7_N calc. for [M + Na]^+^ 354.1523; found 354.1523.

**^2^H**_β_ **(S)‐methyl‐2‐bis(*tert*‐butoxycarbonyl)amino‐4‐oxobutanoate (4)**: To a stirred solution of aldehyde **3** (2.32 g, 7 mmol, 1 eq.) in 40 mL deuterated methanol (MeOD) was added TEA (0.2 mL, 1.4 mmol, 0.2 eq.) under inert conditions. The reaction was stirred for 65 hours at room temperature before evaporating the solvent in vacuo to give the deuterated aldehyde **4** as a yellow oil in quantitative yield (2.33 g).

^1^H NMR (400 MHz, CDCl_3_) *δ*_H_: 9.78 (s, 1H; C*_γ_* HO), 5.50 (s, 1H; C*_α_* H), 3.72 (s, 3H; OCH_3_), 1.49 (s, 18H; Boc_CH3_). ^13^C NMR (101 MHz, CDCl_3_) *δ*_C_: 198.71 (C*_γ_* HO), 170.40 (CO), 151.81 (Boc_CO_), 83.84 (Boc_tBu_), 52.98 (OCH_3_), 52.73 (C*_α_*), 44.57 (qu, C*_β_*), 28.08 (Boc_CH3_). HRMS: *m/z* C_15_H_23_D_2_O_7_N calc. for [M + Na]^+^ 356.1649; found 356.1652.

**^2^H**_β_ **(S)‐methyl‐2‐bis(*tert*‐butoxycarbonyl)amino‐4‐hydroxybutanoate (5)**: Aldehyde **4** (1.45 g, 4.3 mmol, 1 eq.) was dissolved in 30 mL dry methanol in an inert atmosphere and cooled to − 10 °C. To this solution, NaBH_4_ (410 mg, 10.8 mmol, 2.5 eq.) was added and the reaction was proceeded for 15 minutes. The reaction was quenched by adding 30 mL of H_2_O and stirred for another hour at − 10 °C. The crude mixture was extracted three times with ice‐cooled ethyl acetate and the combined organic layers washed with ice‐cooled brine and dried over anhydrous MgSO_4_. The solvent was evaporated in vacuo to give alcohol **5** as a colorless oil in quantitative yield (1.45 g). Despite minor impurities, the product was not further purified due to the instability of the compound.

^1^H NMR (600 MHz, CDCl_3_) *δ*_H_: 4.99 (s, 1H; C*_α_* H), 3.73 (s, 3H; OCH_3_), 3.73 (d, *J = *11.7 Hz, 1H; C*_γ_* H_2a_), 3.58 (d, *J = *11.7 Hz, 1H; C*_γ_* H_2b_), 2.47 (br s, 1H; OH), 1.50 (s, 18H; Boc_CH3_). traces of nondeuterated species: 2.43–2.38 (m), 2.03–1.99 (m). ^13^C NMR (151 MHz, CDCl_3_) *δ*_C_: 171.46 (CO), 152.63 (Boc_CO_), 83.83 (Boc_tBu_), 59.05 (C*_γ_*), 55.50 (C*_α_*), 52.46 (OCH_3_), 32.69 (qu, C*_β_*), 28.11 (Boc_CH3_). HRMS: *m/z* C_15_H_25_O_7_ND_2_ calc. for [M + Na]^+^ 358.1805; found 358.1813.

**^2^H**_β_ **(S)‐methyl‐2‐bis(*tert*‐butoxycarbonyl)amino‐4‐iodobutanoate (6)**: To a stirred solution of alcohol **5** (1.45 g, 4.3 mmol, 1 eq.) in 55 mL dry THF (inert atmosphere) were added sequentially triphenyl phosphine (1.74 g, 6.6 mmol, 1.53 eq.), imidazole (448 mg, 6.6 mmol, 1.53 eq.), and I_2_ (838 mg, 6.6 mmol, 1.53 eq.). The reaction was allowed to proceed at room temperature for 90 minutes until complete conversion was observed by TLC (heptane/ethyl acetate 2:1) and subsequently quenched with 55 mL H_2_O. The crude mixture was extracted three times with ethyl acetate and the combined organic layers washed with brine solution before drying over anhydrous MgSO_4_. The solvent was evaporated in vacuo and the residue purified by flash column chromatography (heptane/ethyl acetate 7:1). Product‐containing fractions were combined to give 1.35 g of the title compound **6** as a light‐yellow oil (71%).

^1^H NMR (400 MHz, CDCl_3_) *δ*_H_: 4.98 (s, 1H; C*_α_* H), 3.73 (s, 3H; OCH_3_), 3.27 (d, *J = *9.9 Hz, 1H; C*_γ_* H_2a_), 3.17 (d, *J = *9.9 Hz, 1H; C*_γ_* H_2b_), 1.51 (s, 18H; Boc_CH3_). ^13^C NMR (101 MHz, CDCl_3_) *δ*_C_: 170.60 (CO), 152.05 (Boc_CO_), 83.69 (Boc_tBu_), 58.54 (C*_α_*), 52.50 (OCH_3_), 31.08 (qu, C*_β_*), 28.15 (Boc_CH3_), 1.58 (C*_γ_*). HRMS: *m/z* C_15_H_24_D_2_O_6_NI calc. for [M + Na]^+^ 468.0823; found 468.0814.

**^2^H**_β_ **(S)‐methyl‐2‐(*tert*‐butoxycarbonyl)amino‐4‐iodobutanoate (7)**: A solution of **6** (3.08 g, 7 mmol, 1 eq.) in 70 mL CH_2_Cl_2_ was cooled to − 10 °C. Subsequently, TFA (1.35 mL, 17.5 mmol, 2.5 eq.) was added slowly. After 50 minutes, another 0.2 mL TFA (2.6 mmol, 0.4 eq.) were added. Complete conversion was indicated by TLC (heptane/ethyl acetate 5:1) after a total reaction time of 1 hour. The crude mixture was concentrated by removing the organic solvent in vacuo and ethyl acetate was added. The mixture was washed three times with a saturated NaHCO_3_ solution. The combined aqueous layers were again extracted with ethyl acetate, and the combined organic layers washed with brine and dried over MgSO_4_. The solvent was evaporated in vacuo to give 2.28 g of the title compound **7** as a white solid (94%).

^1^H NMR (400 MHz, CDCl_3_) *δ*_H_: 5.06 (m, 1H; NH), 4.34 (d, *J = *7.7 Hz, 1H; C*_α_* H), 3.77 (s, 3H; OCH_3_), 3.16 (s, 2H; C*_γ_* H_2_), 1.45 (s, 9H; Boc_CH3_). ^13^C NMR (101 MHz, CDCl_3_) *δ*_C_: 172.16 (CO), 155.43 (Boc_CO_), 80.40 (Boc_tBu_), 54.28 (C*_α_*), 52.89 (OCH_3_), 36.55 (qu, C*_β_*), 28.41 (Boc_CH3_), ‐0.77 (C*_γ_*). HRMS: *m/z* C_10_H_16_D_2_O_4_NI calc. for [M + Na]^+^ 368.0298; found 368.0297, calc. for [2 M + Na]^+^ 713.0704; found 713.0700.

**^13^C_δ_/^2^H_β_/^15^N_ε_ (S)‐methyl‐2‐(*tert*‐butoxycarbonyl)amino‐4‐cyanobutanoate (8)**: To a stirred solution of compound **7** (2.28 g, 6.6 mmol, 1 eq.) in 45 mL dry dimethylformamide (DMF) was added [^13^C^15^N] potassium cyanide (886 mg, 13.2 mmol, 2 eq.) under inert conditions. The reaction mixture was stirred for 16 hours at room temperature before quenching remaining cyanide with saturated NaHCO_3_ solution. The crude solution was concentrated in vacuo. Twenty millilitre ethyl acetate was added and the organic phase washed three times with a saturated NaHCO_3_ solution. The combined aqueous layers were again extracted with ethyl acetate, and combined organic layers washed with brine and dried over MgSO_4_. The solvent was removed in vacuo. The orange oily residue was purified by flash column chromatography (heptane/ethyl acetate 3:1) and product‐containing fractions were combined to give 1.51 g of title compound **8** as colorless oil (93%).

^1^H NMR (400 MHz, CDCl_3_) *δ*_H_: 5.20 (br s, 1H; NH), 4.35 (d, *J = *6.9 Hz, 1H; C*_α_* H), 3.78 (s, 3H; OCH_3_), 2.51–2.32 (m, 2H; C*_γ_* H_2_), 1.44 (s, 9H; Boc_CH3_). ^13^C NMR (101 MHz, CDCl_3_) *δ*_C_: 171.67 (CO), 155.41 (Boc_CO_), 118.84 (d, *J =* 16.9 Hz, ^13^C*_δ_* ^15^N), 80.64 (Boc_tBu_), 52.92 (OCH_3_), 52.43 (C*_α_*), 28.35 (Boc_CH3_), 28.19 (qu, C*_β_*), 13.52 (dd, *J =* 57.1, 2.8 Hz, C*_γ_*). ^15^N NMR (61 MHz, CDCl_3_) *δ*_N_: 246.75 (d, *J = *17.2 Hz, ^15^N*_ε_*). HRMS: *m/z* C_10_^13^CH_16_D_2_O_4_N^15^N calc. for [M + Na]^+^ 269.1288; found 269.1286, calc. for [2M + Na]^+^ 515.2684; found 515.2681.

**^13^C_δ_/^2^H_βγ_/^15^N_ε_ (S)‐2‐(*tert*‐butoxycarbonyl)amino‐4‐cyanobutanoic acid (9)**: A microwave vial was loaded with compound **8** (110 mg, 0.46 mmol, 1 eq.) in 2.5 mL D_2_O. To this solution, DBU (1.1 mL, 7.4 mmol, 18.5 eq.) was added. The reaction vessel was flushed with argon, sealed and placed in a microwave apparatus at 100 °C for 1 hour. The mixture was allowed to reach room temperature and carefully adjusted to pH = 3 using DCl. The aqueous phase was extracted three times with diethyl ether and the combined organic layers were washed with brine solution before drying over anhydrous MgSO_4_. The solvent was evaporated in vacuo to give 87 mg of title compound **9** as a colorless oil (84%). A deuteration grade of 94% was calculated from ^1^H NMR signal integrals.

^1^H NMR (400 MHz, MeOD) *δ*_H_: 4.16 (s, 1H: C*_α_* H), 1.45 (s, 9H; Boc_CH3_). traces of nondeuterated species: 2.54–2.50 (m). ^13^C NMR (101 MHz, MeOD) *δ*_C_: 174.72 (CO), 158.10 (Boc_CO_), 120.31 (d, *J =* 17.4 Hz, ^13^C*_δ_* ^15^N), 80.75 (Boc_tBu_), 53.69 (C*_α_*), 28.67 (Boc_CH3_), 27.97 (qu, C*_β_*), 14.22 (qu, C*_γ_*). ^15^N NMR (61 MHz, CDCl_3_) *δ*_N_: 243.18 (d, *J =* 17.5 Hz, ^15^N*_ε_*). HRMS: *m/z* C_9_^13^CH_12_D_4_O_4_N^15^N calc. for [M‐H]^−^ 233.1292; found 233.1295, calc. for [2M‐H]^−^ 467.2657; found 467.2661.

**^13^C_δ_/^2^H_βγ_/^15^N_ε_ *N*‐(*tert*‐butoxycarbonyl)‐l‐ornithine (10)**: A 10 mL schlenk tube was charged with a solution of **9** (64 mg, 0.27 mmol, 1 eq.) in 5 mL dry methanol. The tube was flushed with argon for 5 minutes before adding 6 mg palladium on carbon (10%). Hydrogen gas was applied using a balloon and the reaction proceeded at room temperature for 16 hours. The mixture was filtered through celite and the solvent evaporated in vacuo to give title compound **10** as a white solid in quantitative yield.

^1^H NMR (600 MHz, MeOD) *δ*_H_: 3.96 (s, 1H; C*_α_* H), 2.93 (d, *J = *142.3 Hz, 2H; ^13^C*_δ_* H_2_), 1.44 (s, 9H; Boc_CH3_). ^13^C NMR (101 MHz, MeOD) *δ*_C_: 176.67 (CO), 158.19 (Boc_CO_), 80.72 (Boc_tBu_), 54.10 (C*_α_*), 39.99 (dt, *J =* 26.1, 6.3 Hz, ^13^C*_δ_*), 28.69 (Boc_CH3_). ^15^N NMR (61 MHz, MeOD) *δ*_N_: 27.90 (d, *J =* 5.0 Hz, ^15^N*_ε_* H_2_). HRMS: *m/z* C_9_^13^CH_16_D_4_O_4_N^15^N calc. for [M + H]^+^ 239.1751; found 239.1746.

**^13^C_δ_/^2^H_βγ_/^15^N_ε_ *N,N,N*‐tris(*tert*‐butoxycarbonyl)‐l‐arginine (11)**: A mixture of starting material **10** (65 mg, 0.27 mmol, 1 eq.) and *N,N’*‐bis‐Boc‐1‐guanyl pyrazole (84 mg, 0.27 mmol, 1 eq.) was dissolved in 5 mL dry methanol under inert conditions. To this solution, diisopropylamine (DIPA) (190 µL, 1.35 mmol, 5 eq.) was added. The reaction was stirred at 50 °C for 3 hours before the solvent was evaporated in vacuo. The crude was purified by flash column chromatography using a gradient of 0 to 10% methanol in CH_2_Cl_2_ as eluent. Product containing fractions were combined to give 76 mg of the title compound **11** as a white solid (58%).

^1^H NMR (400 MHz, CDCl_3_) *δ*_H_: 11.44 (br s, 1H; COOH), 9.15 (br s, 1H; N_η_H), 8.38 (d, *J* = 90.8 Hz, 1H; ^15^N*_ε_* H), 5.37 (s, 1H; NH), 4.27 (s, 1H; C*_α_* H), 3.38 (d, *J* = 139.8 Hz, 2H; ^13^C*_δ_* H_2_), 1.48 (s, 9H; Boc_CH3_), 1.47 (s, 9H; Boc_CH3_), 1.43 (s, 9H; Boc_CH3_). ^13^C NMR (151 MHz, CDCl_3_) *δ*_C_: 163.33 (CO), 156.35 (d, *J =* 24.1 Hz, C_ζ_) 156.27, 155.94, 153.33 (Boc_CO_), 83.36, 80.15, 79.66 (Boc_tBu_), 53.55 (C*_α_*), 40.47 (t, *J =* 9.3 Hz, ^13^C*_δ_*), 29.01 (qu, C*_β_*), 28.48, 28.36, 28.18 (Boc_CH3_), 24.65 (qu, C*_γ_*). ^15^N NMR (61 MHz, CDCl_3_) *δ*_N_: 97.23 (d, *J =* 86.5 Hz, ^15^N*_ε_*). HRMS: *m/z* C_20_^13^CH_34_D_4_O_8_N_3_^15^N calc. for [M + H]^+^ 481.3017; found 481.3008, calc. for [M + Na]^+^ 503.2837; found 503.2828, calc. for [2M + Na]^+^ 983.5781; found 983.5761.

**^13^C_δ_/^2^H_βγ_/^15^N_ε_ l‐arginine (12)**: A solution of **11** (61 mg, 0.13 mmol, 1 eq.) in 3 mL CH_2_Cl_2_ was acidified with 1.5 mL TFA and stirred overnight at room temperature. The solvent was evaporated in vacuo to give title compound **12** as TFA salt in quantitative yield. The product can be transformed in the respective HCl salt by stirring in 0.1 M HCl aqueous solution at room temperature for 1 hour and evaporating the solvent in vacuo. For the overexpression in *E. coli*, the TFA salt was subjected to a DOWEX (50X‐8, H^+^ form) column. After washing with H_2_O, the product was eluted with a 0.2 M NH_4_OH aqueous solution. Product‐containing fractions were combined and lyophilized.

^1^H NMR (600 MHz, D_2_O) *δ*_H_: 4.1 (s, 1H; C*_α_* H), 3.24 (d, *J = *139.9 Hz, 2H; ^13^C*_δ_* H_2_). ^1^H NMR (600 MHz, DMSO‐d_6_) *δ*_H_: 3.02 (d, *J = *138.9 Hz, 2H; ^13^C*_δ_* H_2_), 2.99 (s, 1H; C*_α_* H). ^1^H NMR (600 MHz, MeOD) *δ*_H_: 3.28 (s, 1H; C*_α_* H), 3.17 (d, *J = *135.5 Hz, 2H; ^13^C*_δ_* H_2_). ^13^C NMR (151 MHz, D_2_O) *δ*_C_: 179.25 (CO), 156.74 (d, *J =* 22.1 Hz, C_ζ_), 54.81 (C*_α_*), 40.59 (dt, *J = *10.6, 8.7 Hz, ^13^C*_δ_*), 30.20 (C*_β_*), 23.60 (qu, C*_γ_*). ^13^C NMR (151 MHz, DMSO‐d_6_) *δ*_C_: 41.10 (^13^C*_δ_*). ^13^C NMR (151 MHz, MeOD) *δ*_C_: 42.08 (^13^C*_δ_*). ^15^N NMR (61 MHz, D_2_O) *δ*_N_: 82.99 (dd, *J =* 25.3, 12.6 Hz, ^15^N*_ε_*). HRMS: *m/z* C_5_^13^CH_10_D_4_O_2_N_3_^15^N calc. for [M‐H]^−^ 179.1229; found 179.1301, calc. for [M + Cl]^−^ 215.1066; found 215.1068, calc. for [M + H]^+^ 181.1444; found 181.1444.

### 4.2 Incorporation Studies

**Ubiquitin Overexpression in *E. coli***: Ubiquitin was expressed from a pET‐21a(+) vector, which was transformed into *E. coli* strain BL21(DE3) using the heat shock method. Cells were grown in M9 minimal media (supplemented with 1 g*/*L ^15^NH_4_Cl for uniform ^15^N labeling) starting at OD_600 _= 0.2 from an overnight culture. Polyamines (spermidine or putrescine) or other chosen metabolites (ornithine, guanidinoacetate or GABA) were added when OD_600_ reached 0.3. Isotopically labeled arginine was added at OD_600_ = 0.6. For unlabeled ubiquitin, cells were grown in Luria–Bertani (LB) medium without further additives. Thirty minutes after arginine addition or, in case of the LB culture, at OD_600 _= 0.6, the protein expression was induced with 1 mM IPTG final concentration for 3 hours before harvesting the cells by centrifugation (2 × 15 minutes, 4000 *g*). Pellets were kept at −70 °C until purification.

**Ubiquitin Purification**: The cell pellets were resuspended in 50 mM Tris‐HCl, pH 8.0 (buffer A) supplemented with protease inhibitor (cOmpleteTM, EDTA‐free), lysed by sonication and treated with a heat shock at 70 °C for 10 minutes. The lysate was centrifuged (40 minutes, 30,000 *g*, 4 °C). After buffer exchange with buffer A, the supernatant was further purified by anion exchange chromatography (Cytiva, RessourceTM Q) using a gradient from buffer A to B (buffer A containing 1 M NaCl). Protein‐containing fractions were identified by SDS‐PAGE and subjected to size exclusion chromatography using buffer A as eluent. Protein solutions were concentrated up to 620 µM and kept at − 20 °C.

**Quantification of Incorporation**: From each protein sample, a ^1^H─^13^C HSQC spectrum was recorded. The isotopically labeled arginine‐^13^C_δ_/^1^H_δ_ signals were expected around 43.5 ppm at 3.1 ppm in the proton dimension. Due to the concentrations of the NMR samples ranging from 120 µM to 620 µM, the spectra were acquired with varying numbers of scans and the overall intensity had to be normalized. To this end, we used the (natural ^13^C abundance) methyl signals in the area spanning from 0.4/9.0 ppm to 1.7/28.0 ppm (^1^H/^13^C). We calculated the height ratio between each methyl signal of Arg‐labeled samples and the respective signal in the unlabeled sample and defined the mean of these ratios as the scaling factor for the respective spectrum. The incorporation rate was finally determined using the height ratio of arginine‐^13^C_δ_/^1^H_δ_ peaks between labeled and unlabeled samples. Because R42 showed low‐signal intensity in the unlabeled sample, these peaks were not considered for the calculation.

### 4.3 SH3 binding Studies

**Cell‐Free Expression of SH3**: The third SH3 domain (SH3‐3) of yeast endocytic protein Sla1 was expressed from a pIVEX2.3d vector using a cell‐free expression system. Protocols as introduced by Imbert et al.^[^ ^60^ ^]^ were simplified as follows: 1) The cell extract was produced from *E. coli* BL21(DE3) star transformed to overexpress T7 RNA polymerase, 2) centrifugation speed after lysis was reduced to 15,000 *g*, and 3) maturation and dialysis steps were left out. The protein was expressed in batch mode (1 mL reaction volume) for 6 hours at 25 °C. Exact compositions can be found in the Supporting Information (Tables S2–S4). The reaction mix was centrifuged at 16,000 *g* for 20 minutes at 4 °C and the protein purified from the supernatant on a HisTrap FF affinity column (Cytiva). After loading with 20 mM Tris‐HCl, 500 mM NaCl, 5 mM imidazole, pH 8.0 (buffer C), the column was washed with 10 column volumes of a high salt buffer (buffer C containing 1 M NaCl) before eluting the protein using buffer D (buffer C containing 300 mM imidazole). Purity was confirmed by SDS‐PAGE and protein solutions concentrated up to 2 mg/mL and kept at − 20 °C.

**Titration Experiments**: The buffer of Sla1 SH3‐3 was exchanged to 50 mM Tris‐HCl, pH 8.0 (buffer A). A 70 µM arginine‐labeled ubiquitin sample was titrated with increasing amounts of Sla1 SH3‐3 solution at 25 °C. To allow direct comparison of peak intensities, the ubiquitin concentration was adjusted to stay at 70 µM in each sample. The titration was monitored by acquiring ^1^H–^13^C HSQC spectra and computing the chemical shift perturbations of arginine‐^13^C_δ_/^1^H_δ_ peaks. As a control experiment, a ^1^H–^15^N SOFAST experiment was performed of each sample and the chemical shift perturbations of the backbone signals were compared to literature.

### 4.4 NMR Spectroscopy

NMR ^1^H, ^13^C, ^15^N spectra of organic small molecules were obtained on a Bruker BioSpin AV III HD 700, Bruker BioSpin AV III 600 or Bruker BioSpin AV NEO 400. Chemical shifts were reported in ppm according to tetramethylsilane using the solvent residual signal as an internal reference (^1^H/^13^C: 7.26/77.16 (CDCl_3_); 4.79 (D_2_O); 2.50/39.52 (DMSO‐d_6_); 3.31/49.00 (MeOD)). Coupling constants (*J*) were given in Hertz and were averaged. Resonance multiplicity was described as s (singlet), d (doublet), t (triplet), q (quartet), qu (quintet), m (multiplet), br (broad signal), dd (doublet of doublets), or td (triplet of doublets). Carbon and nitrogen spectra were acquired with proton decoupling.

Protein NMR spectra were obtained on an 800 MHz Avance NEO spectrometer equipped with a TCI ^1^H/^13^C/^15^N 5 mm cryoprobe, using 3 mm sample tubes, at a sample temperature of 298 K. The ^1^H–^13^C correlation spectra used for quantifying the extent of labeling and for titration experiments used a sensitivity‐enhanced HSQC pulse sequence (implemented in NMRlib^[^ ^61^ ^]^) with a ^13^C spectral width of 70 ppm and 448 increments (maximum evolution time 17.3 ms). Experimental durations for the ^1^H–^13^C experiments ranged from 2 to 24 hours, adapted to the concentration of the samples.

Backbone ^1^H–^15^N spectra were obtained with a SOFAST‐HMQC experiment^[^ ^62^ ^]^ using a recycle delay of 0.2 s, and 360 increments in the ^15^N dimension (spectral width 36 ppm, maximum evolution time 62 ms), with 16 scans per increment, resulting in 28 minutes total experimental duration.

The H_δ_─C_δ_─N_ε_ correlation experiment (Figure 3) used a pulse sequence originally written for H_α_–C_α_–N correlation spectra^[^ ^63^ ^]^ (implemented in the Bruker library as hcangp3d), adapted by setting the ^13^C (^15^N) carrier position to 40 ppm (85 ppm). The 3D experiment used 90 (80) increments in the ^13^C (^15^N) dimensions, with 10 ppm in each of the two dimensions, leading to maximum evolution times in the two dimensions of 22 and 49 ms, respectively. Nonuniform sampling with 12.3% sampled points was used. The spectral width in the ^1^H dimension was 15.6 ppm and the acquisition was 82 ms (2048 points). With 16 scans per increment and a recycle delay of 1 s, the total duration of the 3D spectrum was 4 hours and 42 minutes. The 2D ^1^H–^15^N version of the experiment also used a 10 ppm spectral width in ^15^N with 80 increments (maximum evolution time 49 ms). Eighty scans per increment were used, and a recycle delay of 1 second, resulting in a total experiment duration of 2 hours and 8 minutes.

## Supporting Information

Further information on the optimization of step (i), the incorporation studies with polyamines and metabolites, and the cell free expression system, as well as NMR spectra of organic compounds can be found in the Supporting Information.

## Conflict of Interest

The authors declare no conflict of interest.

## Supporting information



Supporting Information

## Data Availability

The data that support the findings of this study are available in the supplementary material of this article.
