# Supplementary material for: Synthesis of Selectively 13C/2H/15N‐ Labeled Arginine to Probe Protein Conformation and Interaction by NMR Spectroscopy
Source: Chemistry. 2025 Apr 3;31(24):e202500408. doi: 10.1002/chem.202500408 (PMC12043044; doi:10.1002/chem.202500408)

Synthesis of Selectively $^{13}C/^{2}H/^{15}N$-Labelled Arginine to Probe Protein Conformation and Interaction by NMR Spectroscopy

Darja I. Rohden,[a,b,c] Giorgia Toscano,[a,c] Paul Schanda,*[b] Roman J. Lichtenecker*[a,d]

[a] D. I. Rohden, G. Toscano, Dr. R. J. Lichtenecker
University of Vienna, Faculty of Chemistry, Institute of Organic Chemistry, Währinger Str. 38, 1090 Vienna, Austria
E-mail: roman.lichtenecker@univie.ac.at

[b] D. I. Rohden, Prof. Dr. P. Schanda
Institute of Science and Technology Austria, Am Campus 1, 3400 Klosterneuburg, Austria
E-mail: paul.schanda@ist.ac.at

[c] D. I. Rohden, G. Toscano
University of Vienna, Vienna Doctoral School in Chemistry (DoSChem), Währinger Str. 42, 1090 Vienna, Austria

[d] Dr. R. J. Lichtenecker
MAG-LAB, Karl-Farkas Gasse 22, 1030 Vienna

Contents

1 Optimization to yield $^{13}C_{\delta}/^{2}H_{\beta,\gamma}/^{15}N_{\varepsilon}$ (S)-2-(*tert*-butoxycarbonyl)amino-4-cyanobutanoic acid (9) 2

2 Incorporation Study 3

3 Conditions for the cell free expression of SH3 4

4 NMR spectra 5

1 Optimization to yield $^{13}C_{\delta}/^{2}H_{\beta,\gamma}/^{15}N_{\varepsilon}$ (S)-2-(*tert*-butoxycarbonyl)amino-4-cyanobutanoic acid (9)

Each reaction was performed in a scale of 50 mg (0.2 mmol) using non-labelled starting material. For reactions 1-6 (table S1), the substrate was dissolved in a mixture of D_2O (0.4 mL) and either dioxane or THF (0.2 mL) before adding triethylamine (0.3 mL, 2.1 mmol, 10 eq), and if indicated LiOH*H_2O or LiCl (13 mg, 0.3 mmol, 1.5 eq). For reactions 7-11, the substrate was dissolved in D_2O (0.45 mL) and dioxane (0.25 mL) before adding DBU (0.35 mL, 2.3 mmol, 11 eq). For reactions 12-16, the volume of D_2O and DBU were increased to 0.9 mL and 0.5 mL, respectively. Reaction 17 was performed without cosolvent but with an adjusted volume of D_2O to maintain the substrate concentration. After the indicated reaction time, the crude was carefully adjusted to pH = 3 using DCl. The aqueous phase was extracted three times with ethyl acetate and the combined organic layers washed with brine solution before drying over anhydrous $MgSO_4$. The solvent was evaporated *in vacuo* and the deuteration determined by NMR.

Table S1: Screening of reaction conditions for step (i): Deuteration and hydrolysis towards **9**.

	Conc.[M][a]	Cosolvent (vol%)	Base	Temp.[°C]	Time[h]	O.D.[%][b]
1*	0.23	Dioxane (20)	TEA + LiOH	rt	18	0
2	0.23	Dioxane (20)	TEA + LiCl	rt	18	0
3	0.23	Dioxane (20)	TEA	rt	96	0
4	0.23	THF (20)	TEA	rt	18	0
5	0.23	THF (20)	TEA	70	1	0
6	0.20	Dioxane (24)	DBU	rt	18	3
7	0.20	Dioxane (24)	DBU	100	2.5	54
8*	0.20	Dioxane (24)	DBU	100	18	50
reactions performed in microwave:						
9	0.20	Dioxane (24)	DBU	100	1	82
10[c]*	0.20	Dioxane (24)	DBU	100-150	1+0.75	76
11	0.20	Dioxane (24)	DBU	100	1.5	79
12	0.13	Dioxane (15)	DBU	100	1	91
13	0.13	Dioxane (15)	DBU	100	3	92
14	0.06	Dioxane (15)	DBU	100	1	60
15	0.06	THF (15)	DBU	100	1	87
16	0.13	THF (15)	DBU	100	1	60
17	0.13	-	DBU	100	1	94

* Side product observed by MS.

[a] Concentration in total reaction volume. [b] Observed deuteration determined by NMR. [c] The product was subjected to a second reaction at 150 °C which led to the formation of side product.

2 Incorporation Study

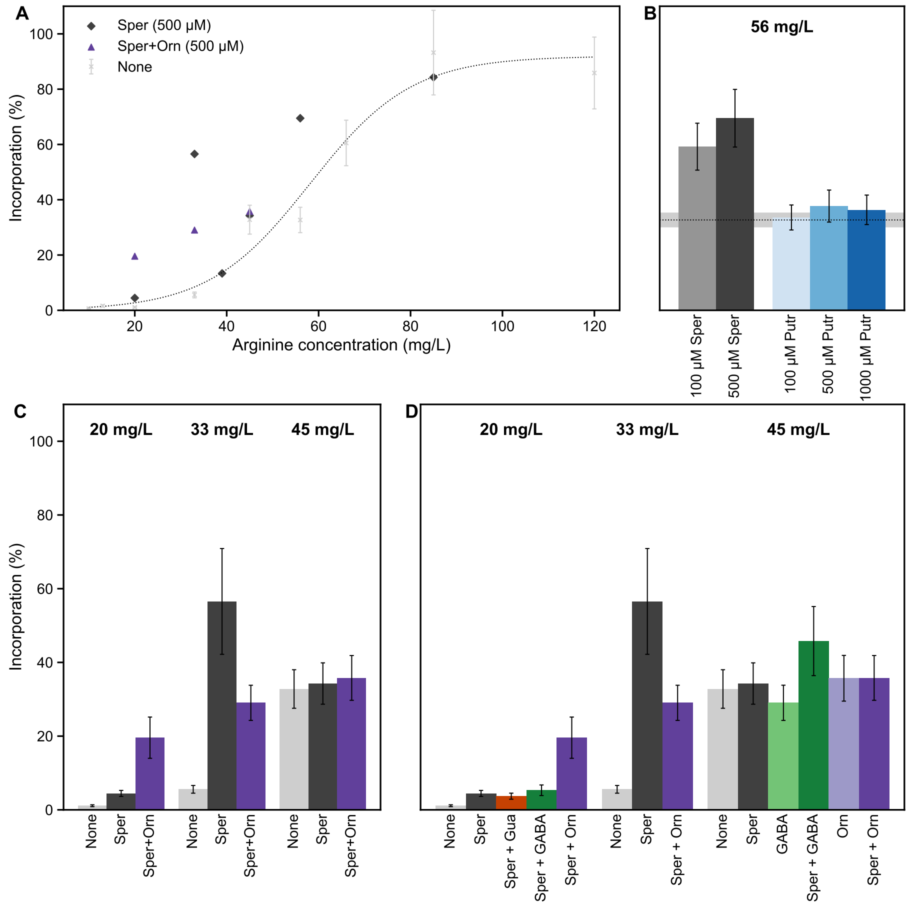

Figure S1: Extended arginine incorporation study. **A** Labelled arginine concentration vs. incorporation rate with or without addition of 500 μM spermidine and ornithine. Dotted line represents sigmoidal fit of the samples without any additives. **B-D** Arginine incorporation at the indicated concentrations of arginine (in bold) with or without addition of polyamines and metabolites. If not stated otherwise, an additive concentration of 500 μM was used. **B** Dotted line represents the incorporation rate without any additive including the standard deviation (grey area).
The data point at 33 mg/L arginine with 500 μM spermidine was not reproducible and is considered an outlier.
Abbreviations: Orn - ornithine; GABA - gamma-aminobutyric acid; Gua - guanidiniumacetate; Putr - putrescine; Sper - spermidine.

3 Conditions for the cell free expression of SH3

Table S2: Composition of cell free reaction

	Final conc.	Volume [μL]
10x buffer	1x	100
AA mix (15 mM)	1 mM	66.67
Creatine phosphate (1 M)	80 mM	80
Potassium glutamate (4 M)	208 mM	52
Magnesium acetate (1.07 M)	16 mM	9.72
Creatine kinase (10 mg/mL)	0.25 mg/mL	25
Plasmid (1000 ng/μL]	10 ng/μL	10
tRNA (17.5 mg/mL	0.175 mg/mL	10
S15 Extract	-	400
milliQ	-	246.61
Final Volume		1000

Table S3: Composition of 10x buffer for cell free reaction

	Final conc. [mM]	Volume [μL]
rCTP (100 mM)	0.8	80
rGTP (100 mM)	0.8	80
rUTP (100 mM)	0.8	80
ATP (100 mM)	1.2	120
HEPES (2 M)	55	275
Folinic Acid (10 mM)	0.068	68
cAMP (100 mM)	0.64	64
DTT (1 M)	3.4	34
Spermidine (850 mM)	2	23.5
NH_4OAc(9.2 M)	27.5	29.9
milliQ	-	145.6
Final Volume		1000

The 10x buffer was stored at –80 °C.

Table S4: Composition of amino acid mix for cell free reaction

	Final conc. [mM]	Volume [μL]
AA in 1 M HCl[a]	15	24
AA in 1 M KOH[b]	15	24
AA in H_2O[c]	15	24
milliQ	-	8
Final Volume		80

The amino acid stocks consist of 50 mM of the following amino acids:
[a] Asn, Asp, Cys, Gln, Glu, Leu, Met, Trp, Tyr
[b] Ile, Phe
[c] Ala, Arg, Gly, His, Lys, Pro, Ser, Thr, Val

4 NMR spectra

Dimethyl *N*-(*tert*-butoxycarbonyl)-L-aspartate (1)

^{1}H NMR (400 MHz, $CDCl_3$)

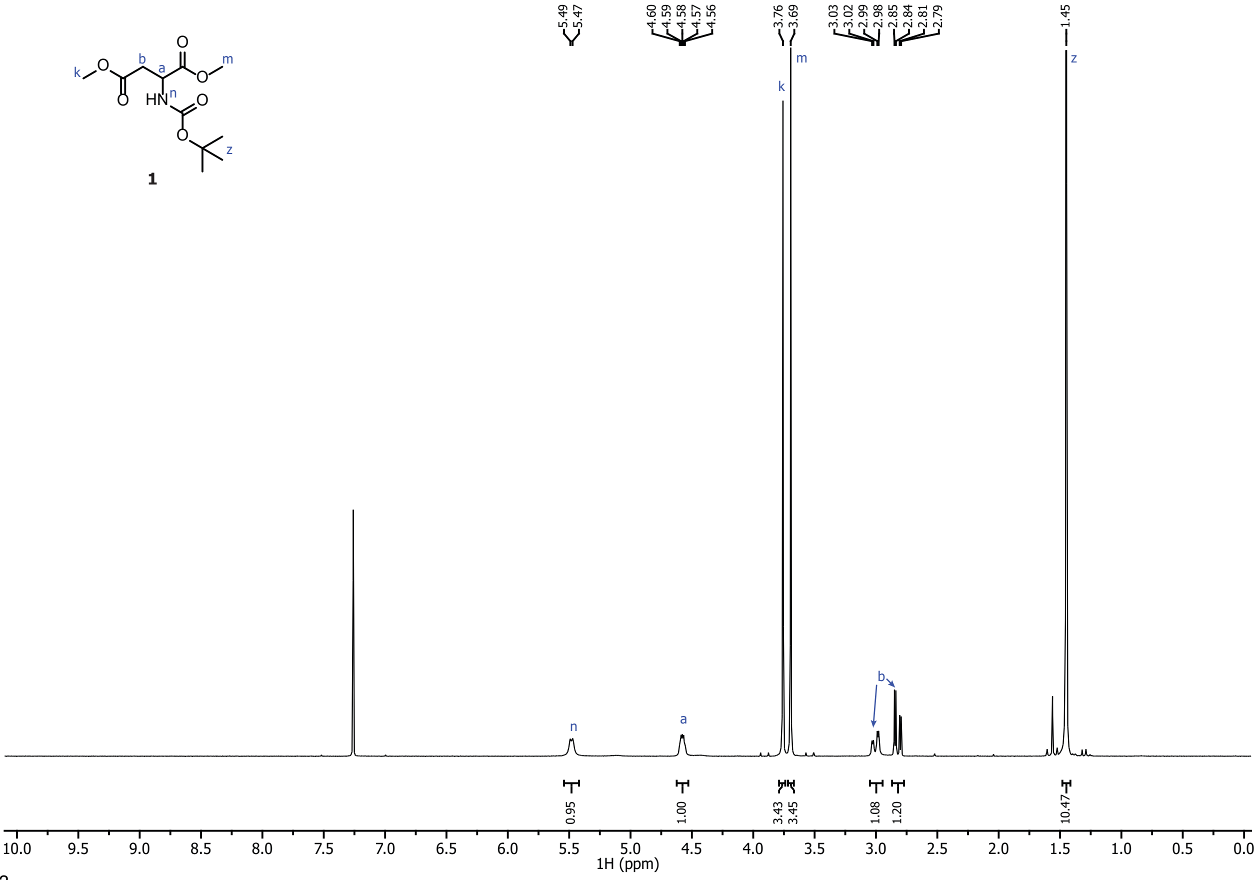

^{13}C NMR (101 MHz, $CDCl_3$)

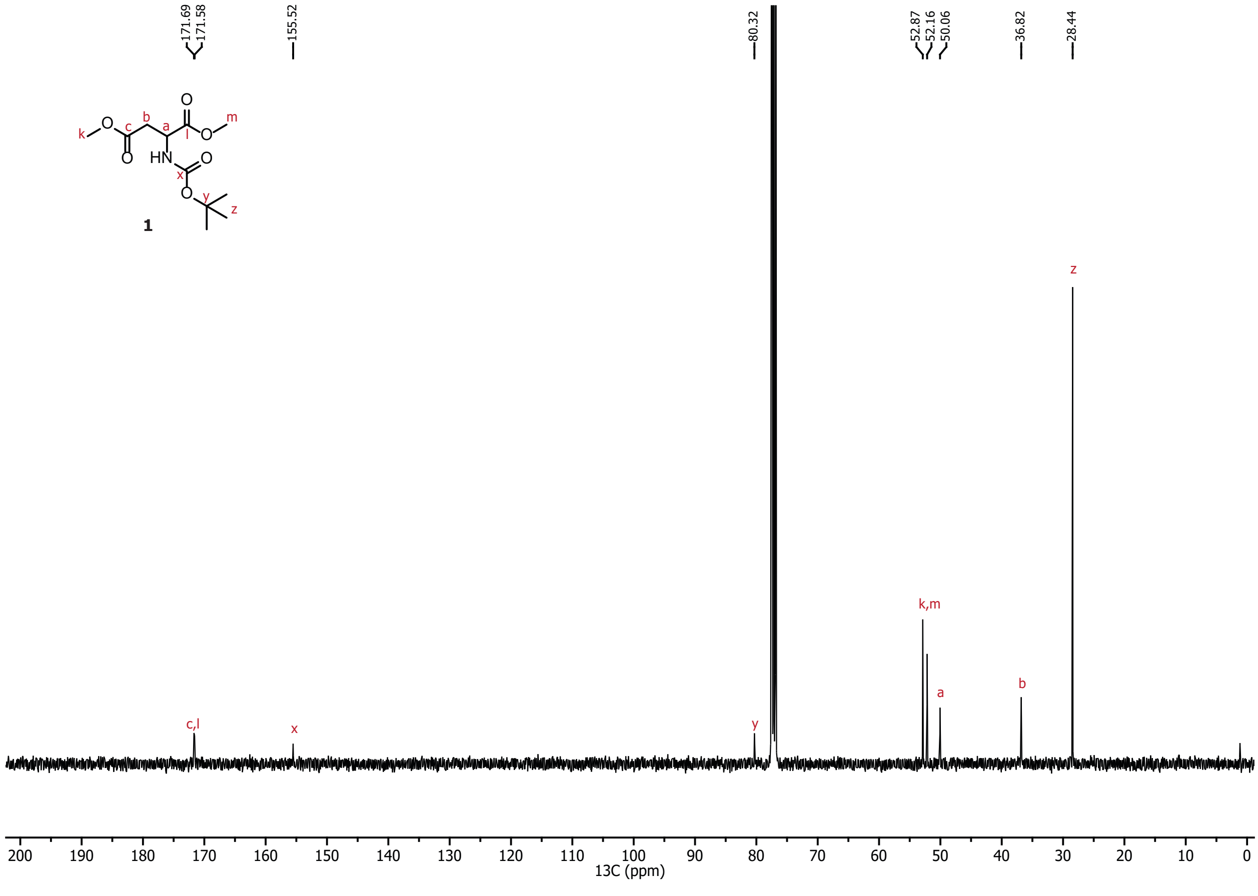

Dimethyl *N,N*-bis(*tert*-butoxycarbonyl)-L-aspartate (2)

^{1}H NMR (700 MHz, $CDCl_3$)

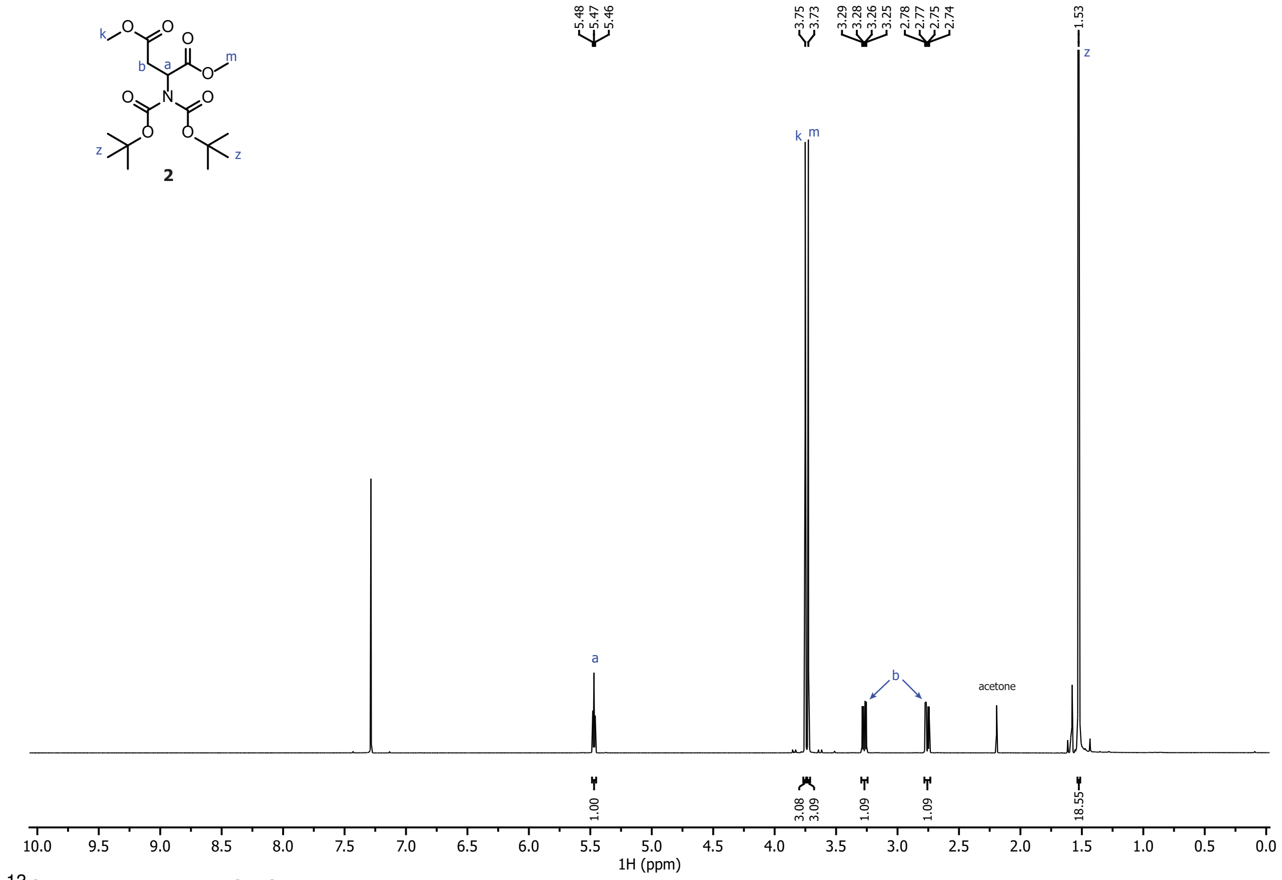

^{13}C NMR (176 MHz, $CDCl_3$)

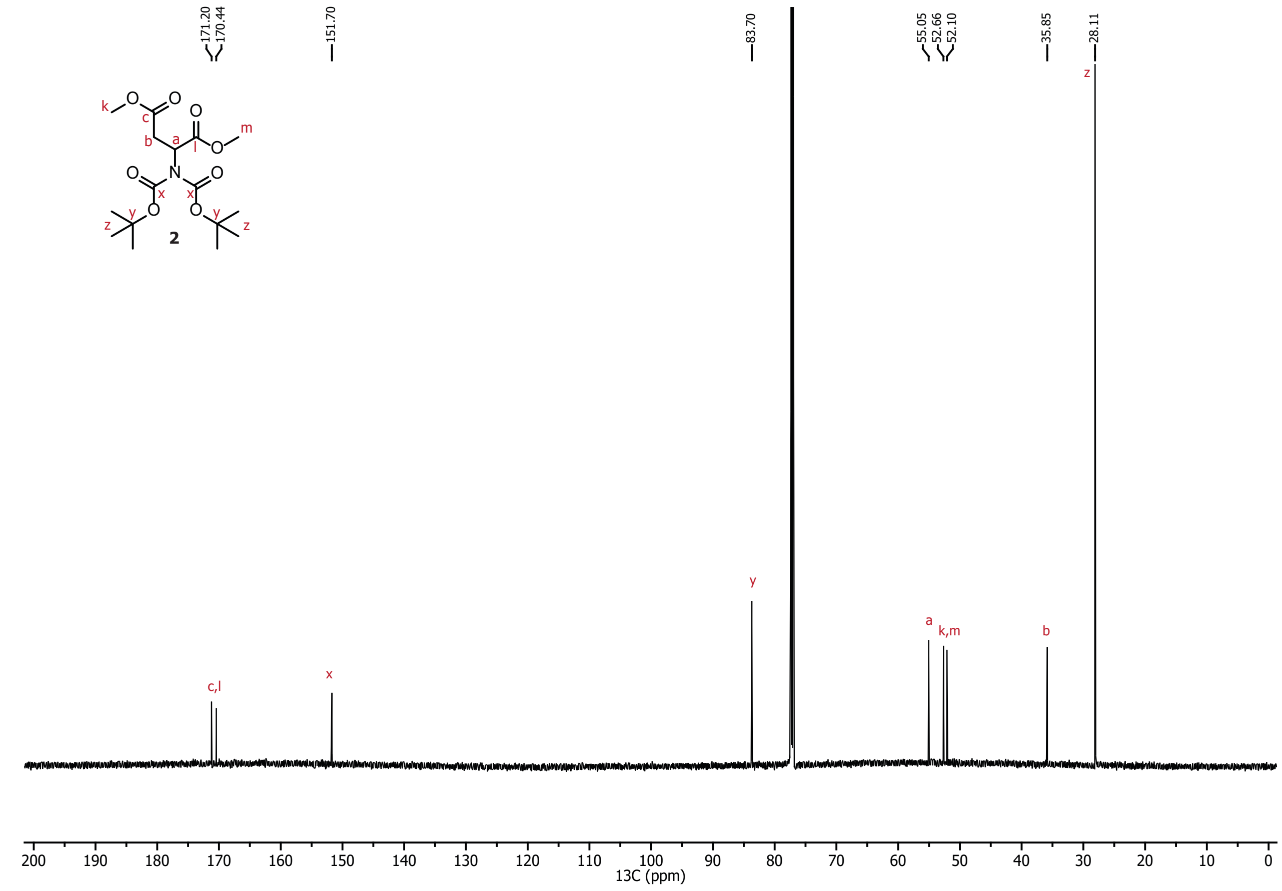

(S)-methyl-2-bis(*tert*-butoxycarbonyl)amino-4-oxobutanoate (3)

^{1}H NMR (700 MHz, $CDCl_3$)

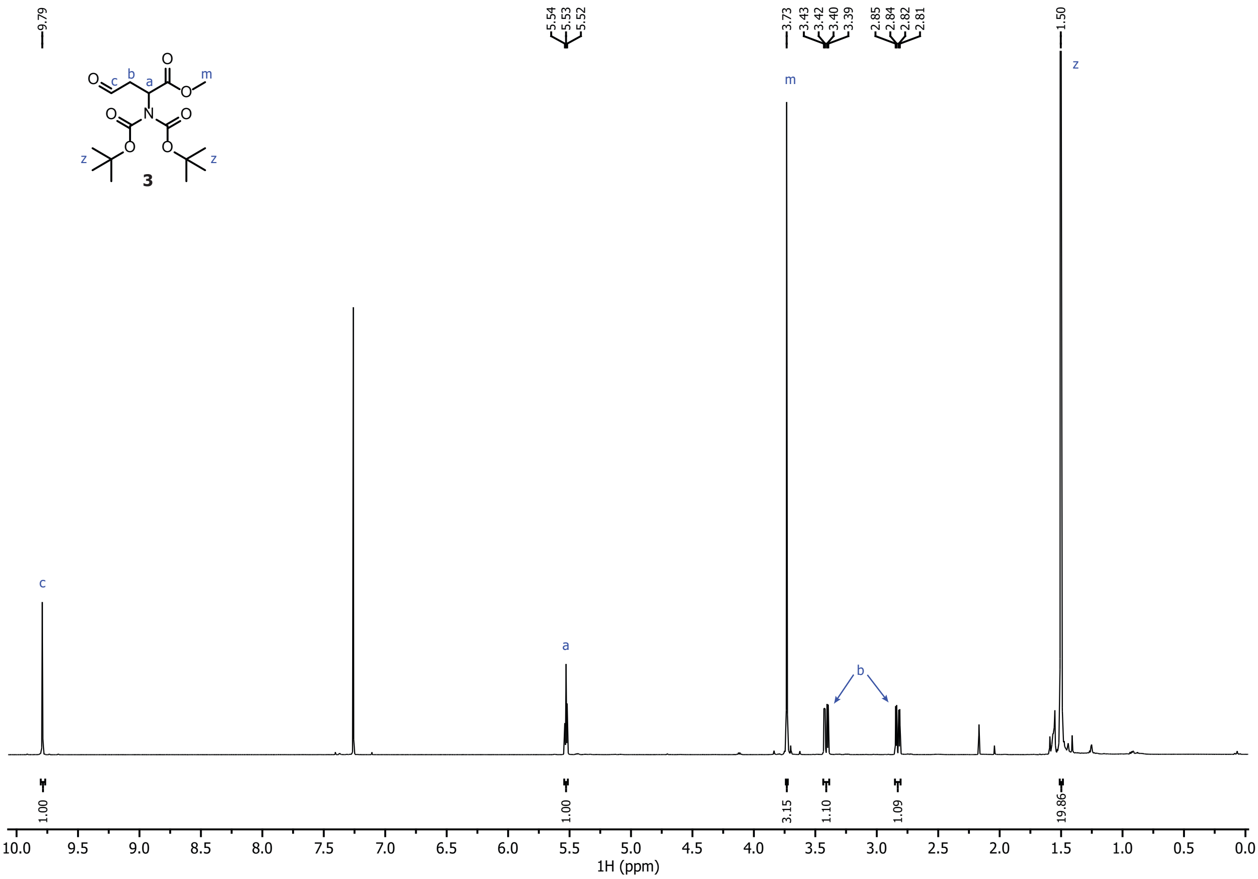

^{13}C NMR (176 MHz, $CDCl_3$)

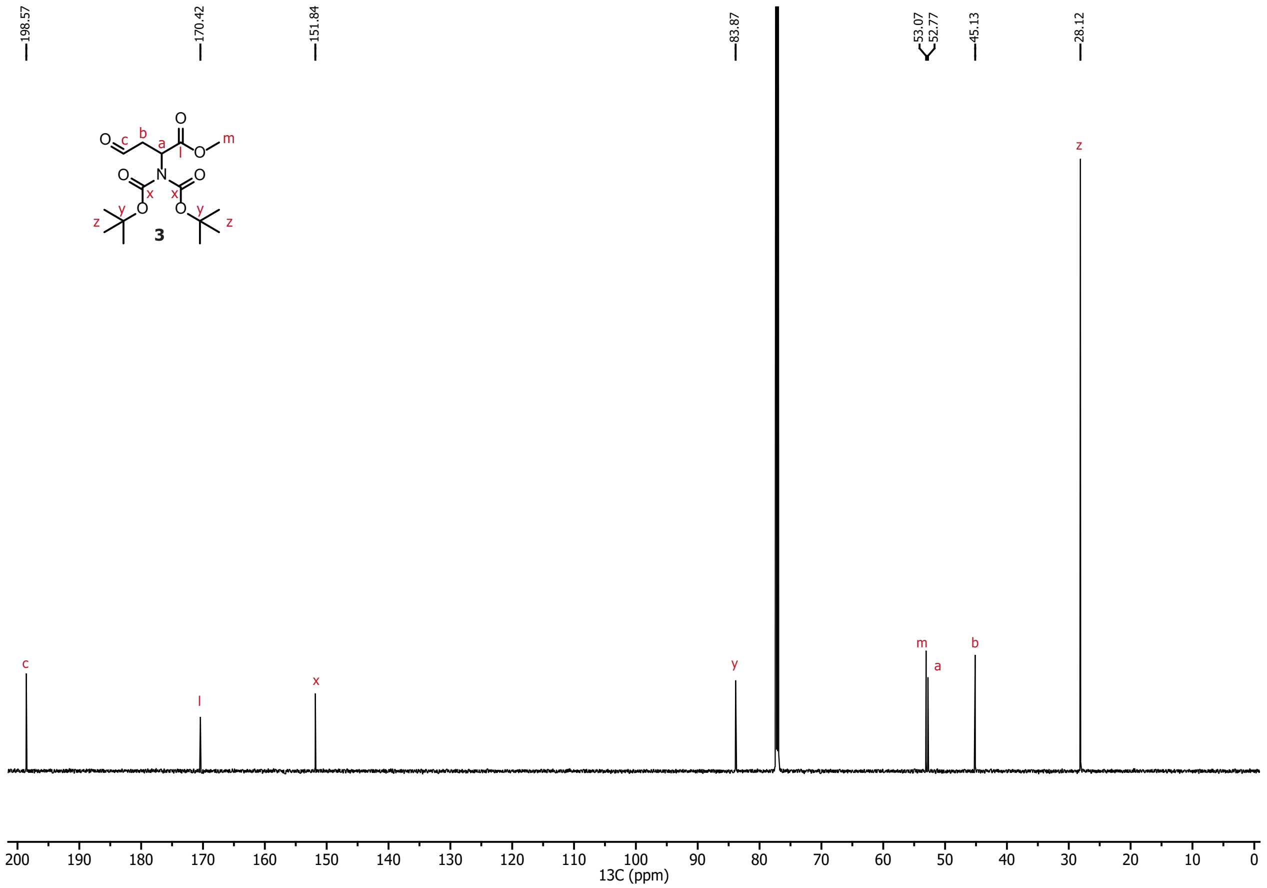

$^{2}H_{\beta}$ (S)-methyl-2-bis(*tert*-butoxycarbonyl)amino-4-oxobutanoate (4)

^{1}H NMR (400 MHz, $CDCl_3$)

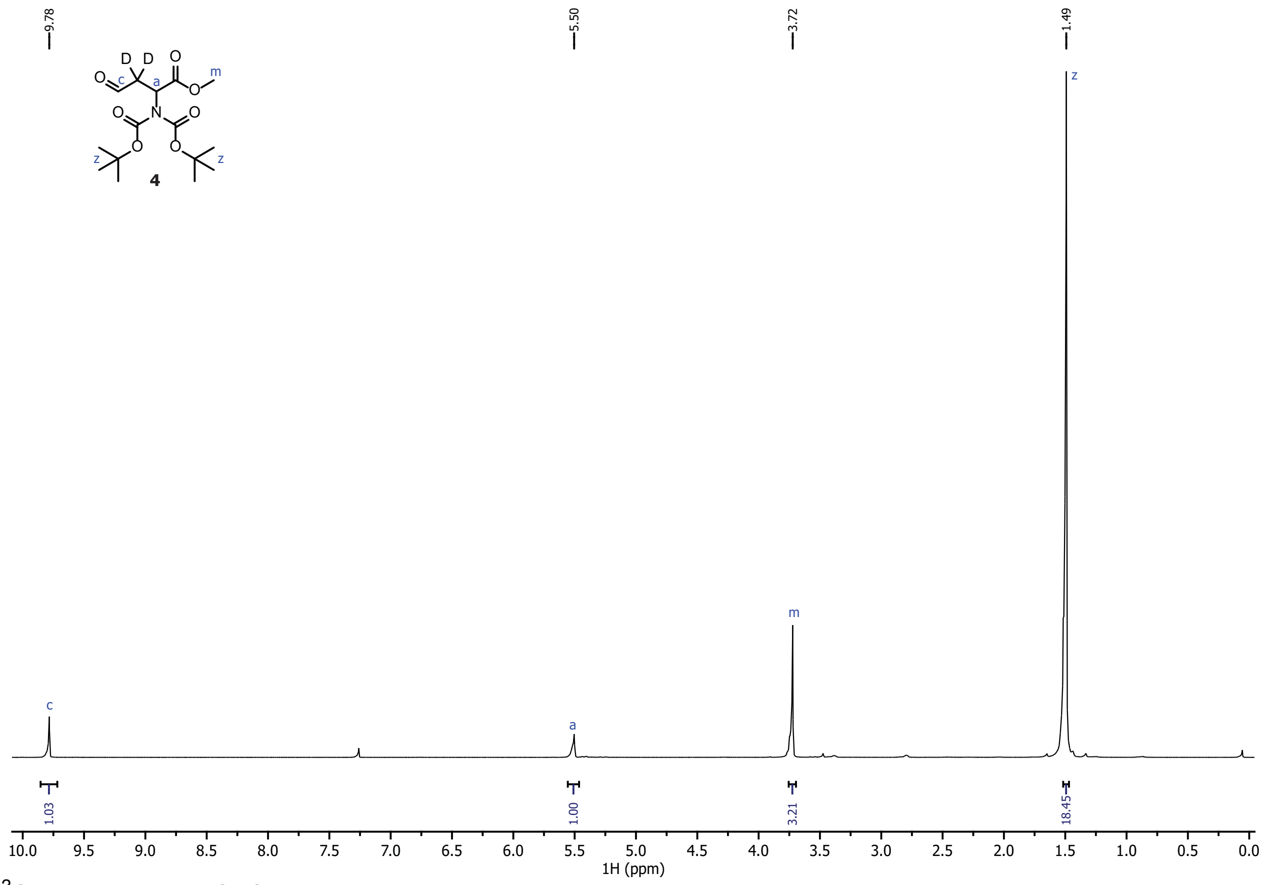

^{13}C NMR (101 MHz, $CDCl_3$)

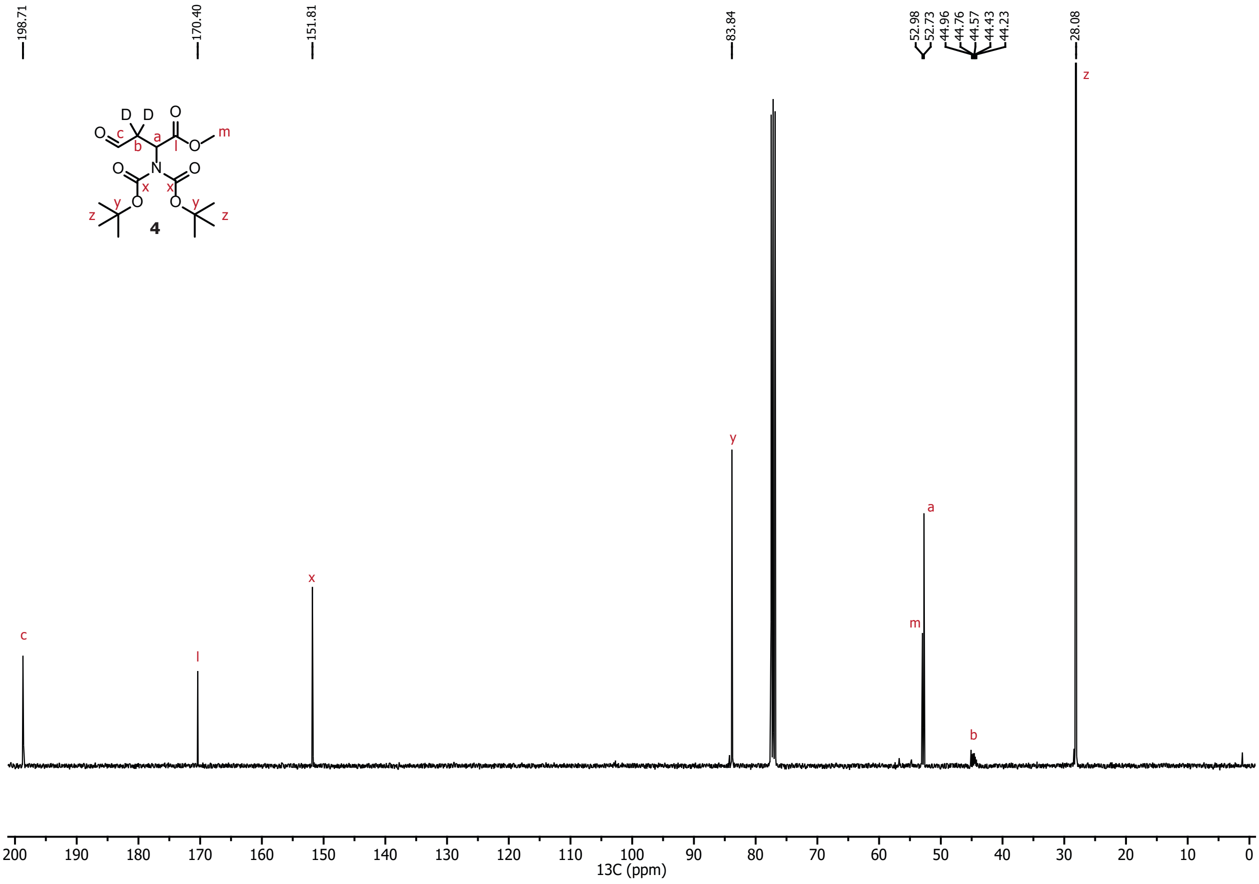

$^{2}H_{\beta}$ (S)-methyl-2-bis(*tert*-butoxycarbonyl)amino-4-hydroxybutanoate (5)

^{1}H NMR (600 MHz, $CDCl_3$)

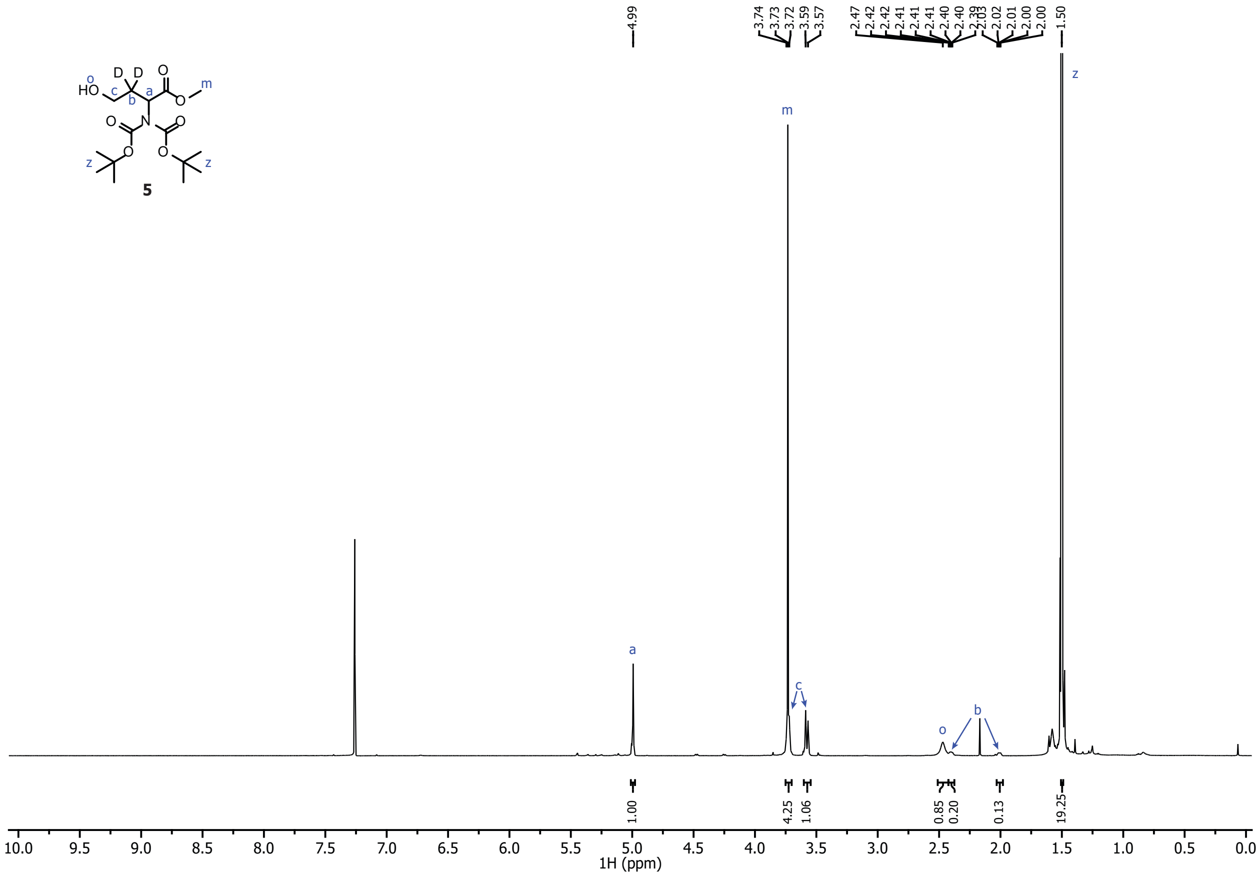

^{13}C NMR (151 MHz, $CDCl_3$)

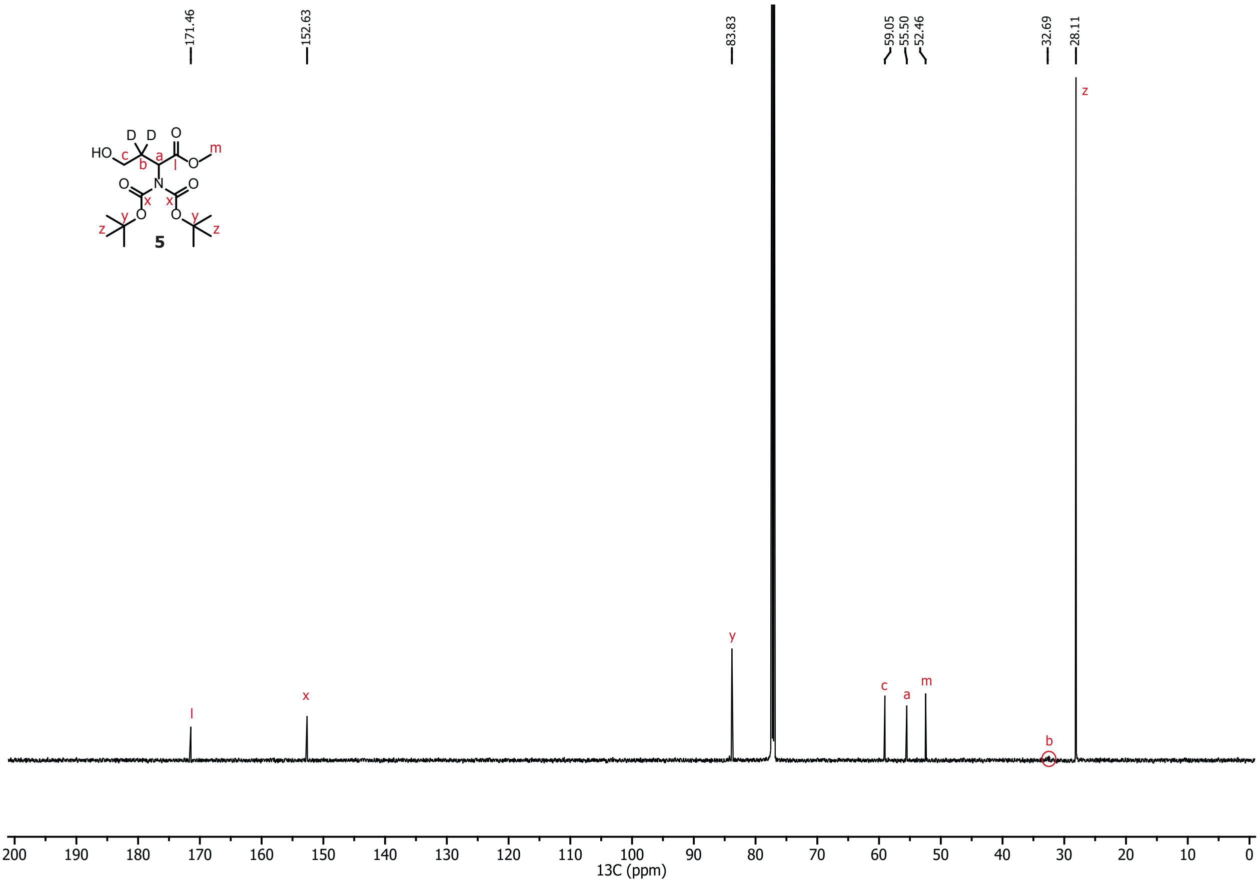

$^{2}H_{\beta}$ (S)-methyl-2-bis(*tert*-butoxycarbonyl)amino-4-iodobutanoate (6)

^{1}H NMR (400 MHz, $CDCl_3$)

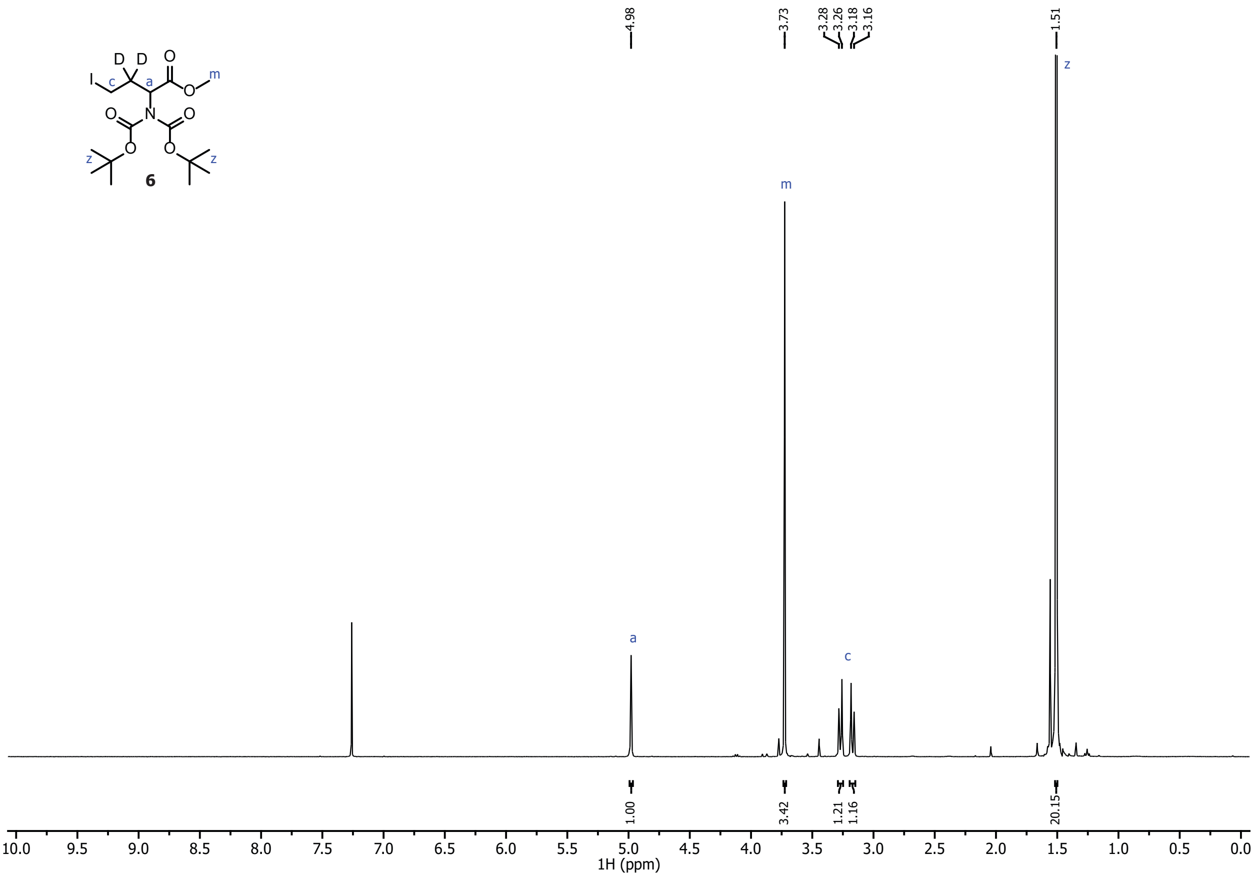

^{13}C NMR (101 MHz, $CDCl_3$)

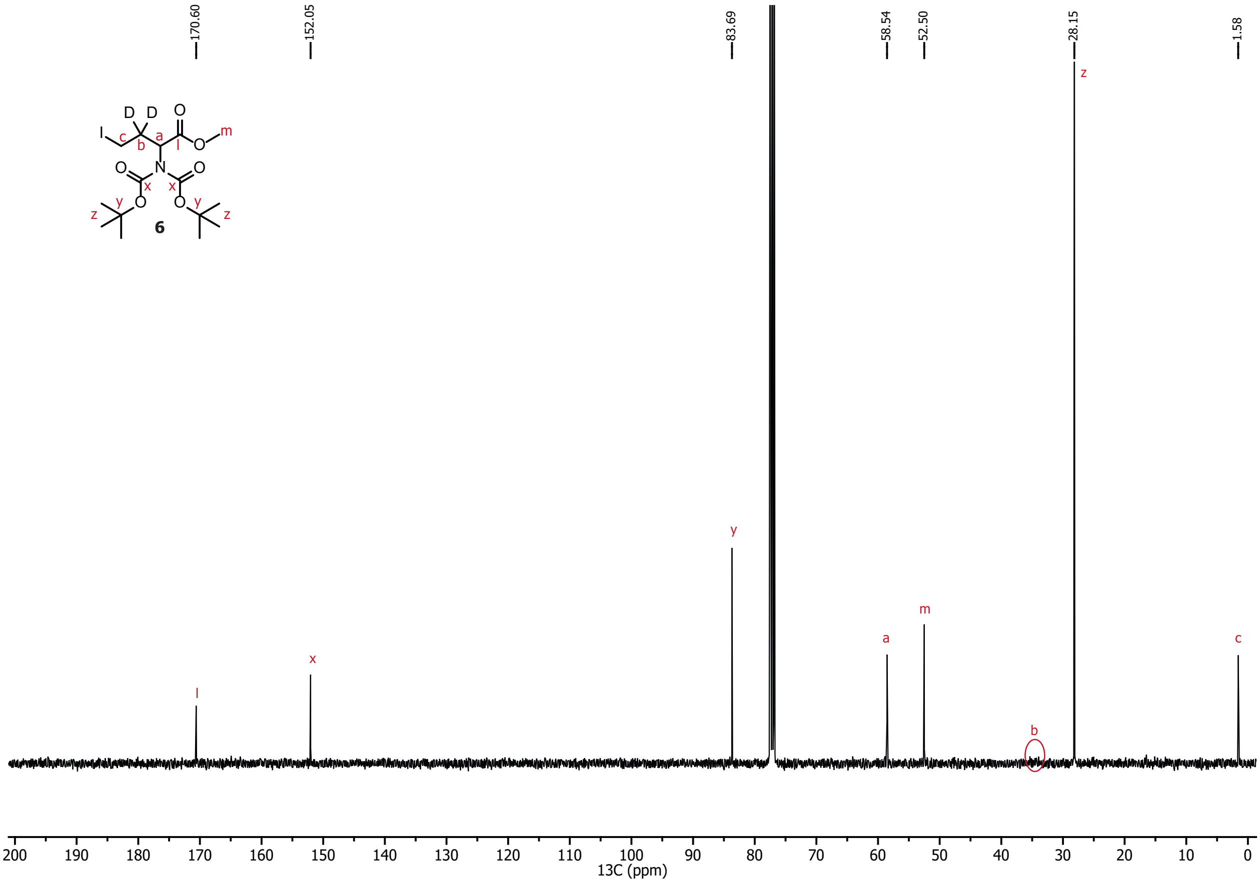

$^{2}H_{\beta}$ (S)-methyl-2-(*tert*-butoxycarbonyl)amino-4-iodo-butanoate (7)

^{1}H NMR (400 MHz, $CDCl_3$)

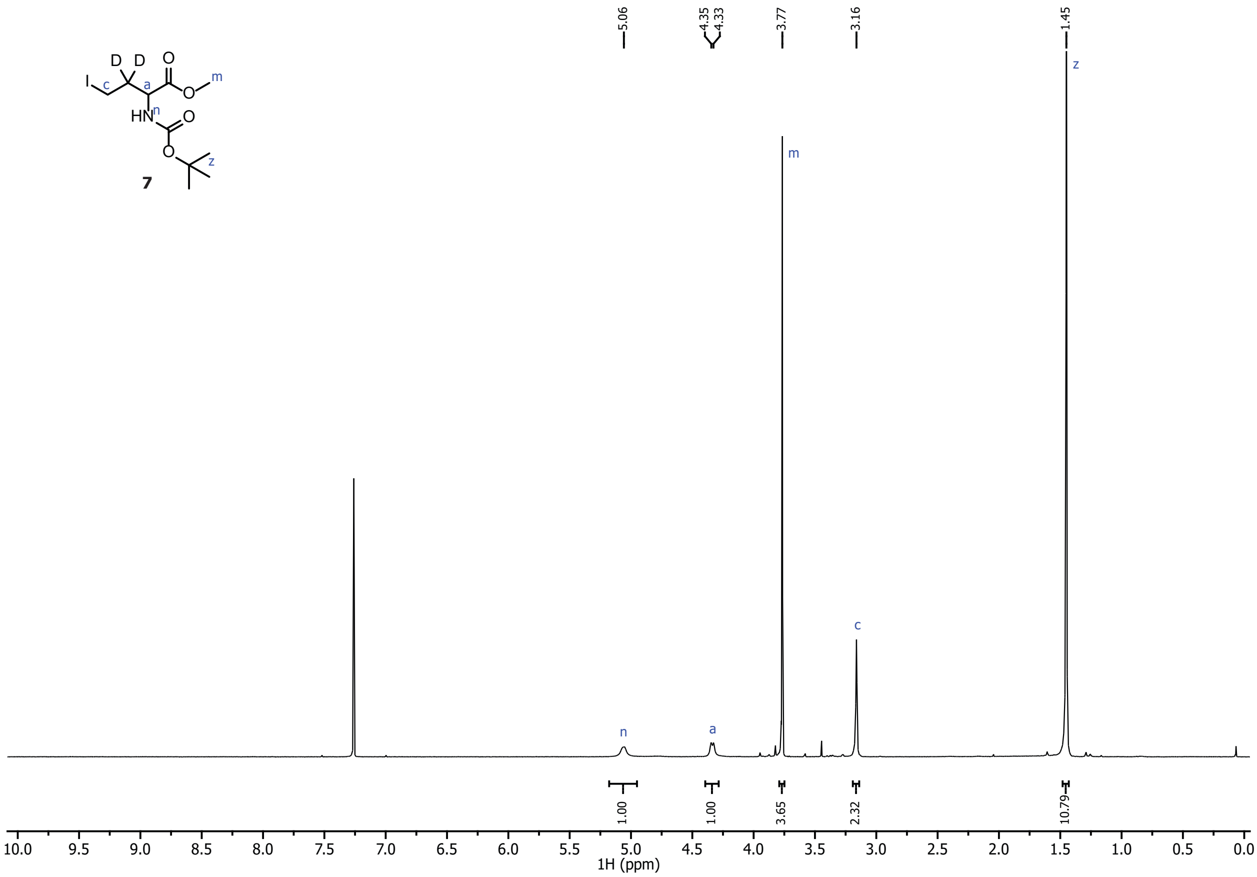

^{13}C NMR (101 MHz, $CDCl_3$)

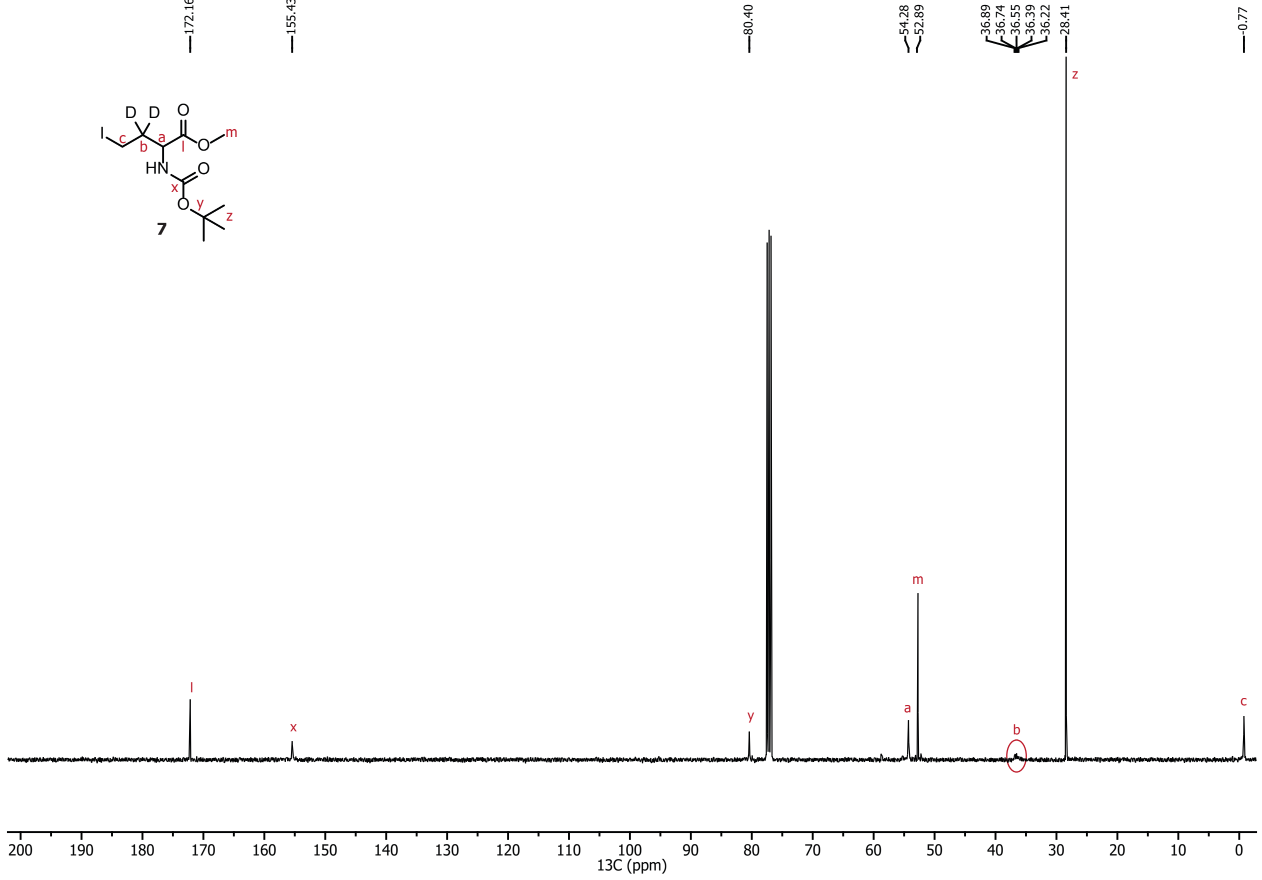

$^{13}C_\delta$/$^{2}H_\beta$/$^{15}N_\varepsilon$ (S)-methyl-2-(*tert*-butoxycarbonyl)-amino-4-cyanobutanoate (8)

^{1}H NMR (400 MHz, $CDCl_3$)

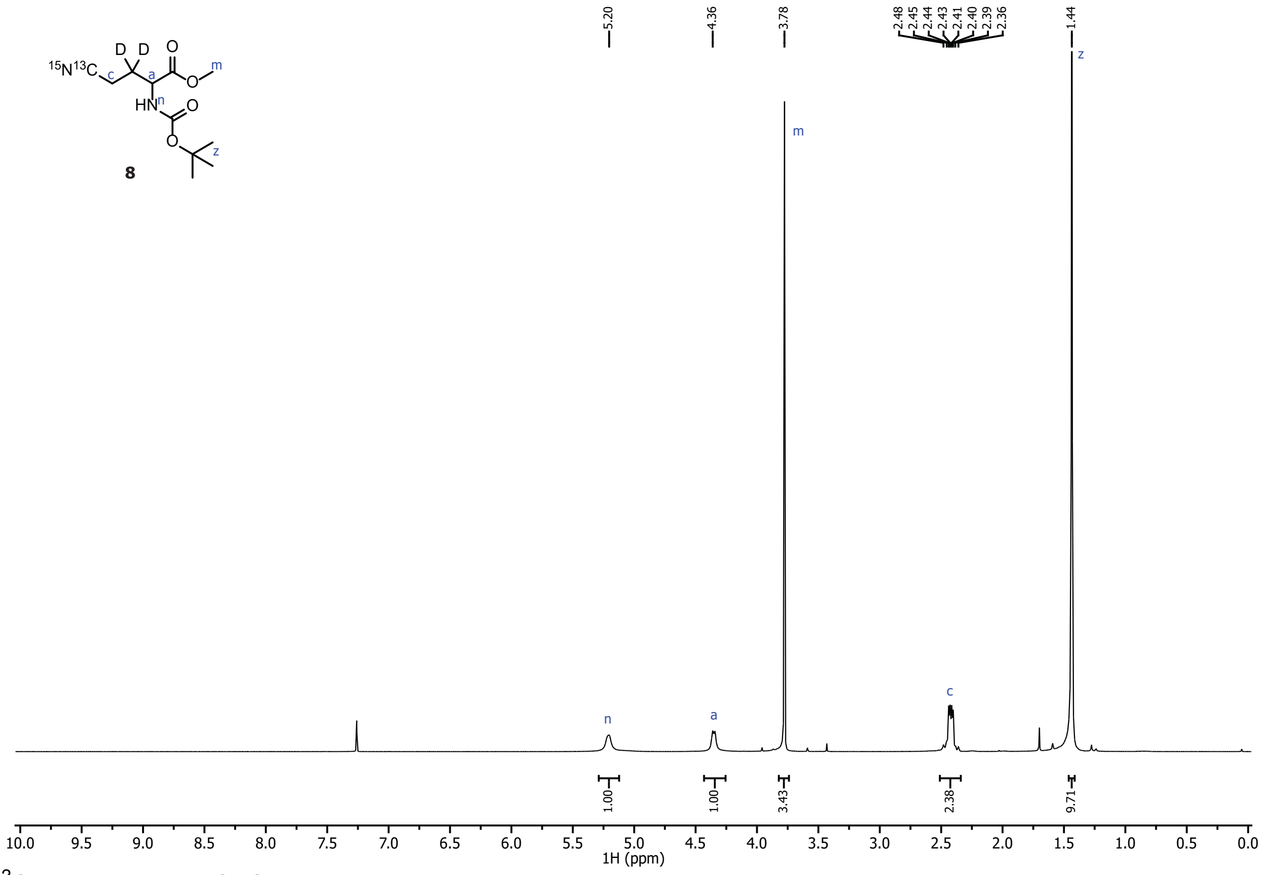

^{13}C NMR (101 MHz, $CDCl_3$)

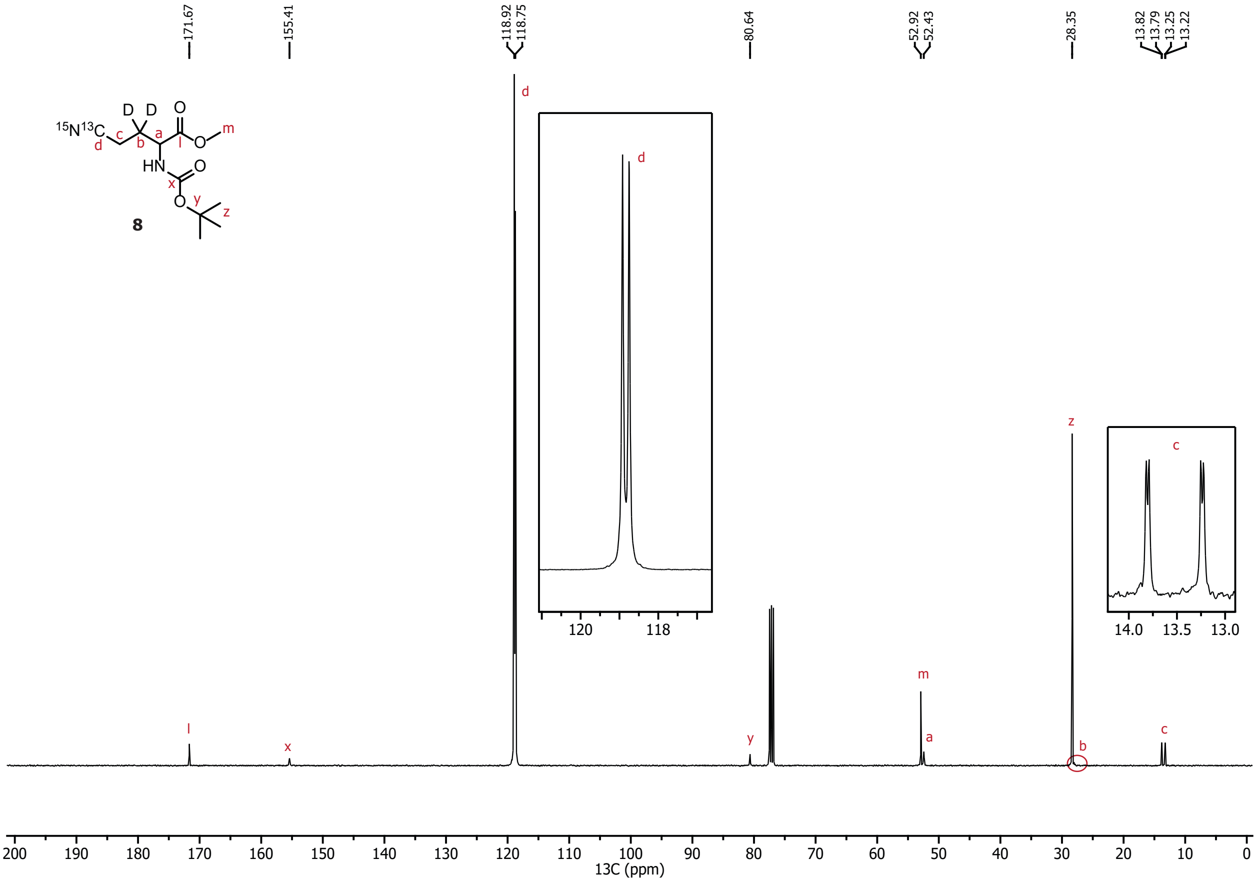

^{15}N NMR (61 MHz, $CDCl_3$)

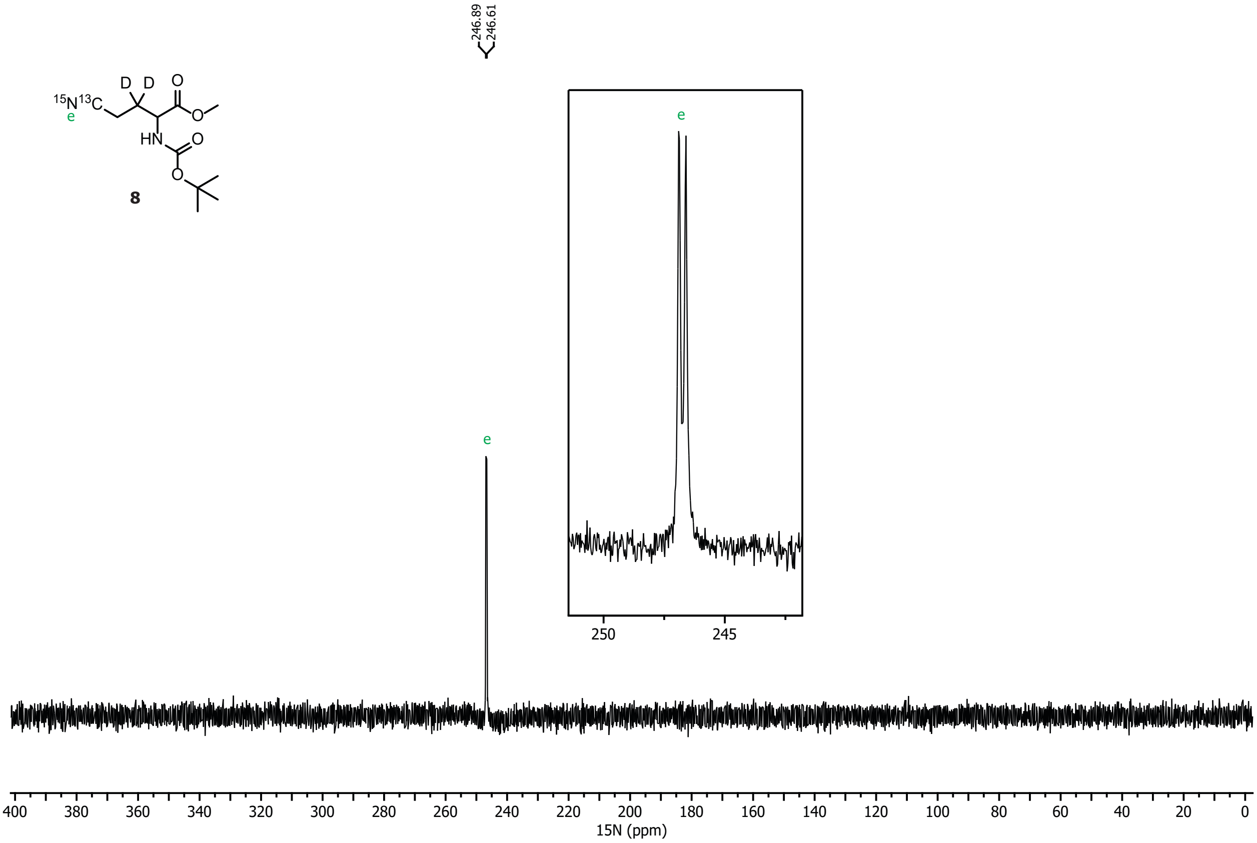

^{13}C$_\delta$/^{2}H$_{\beta,\gamma}$/^{15}N$_\varepsilon$ (S)-2-(*tert*-butoxycarbonyl)amino-4-cyanobutanoic acid (9)

^{1}H NMR (400 MHz, MeOD)

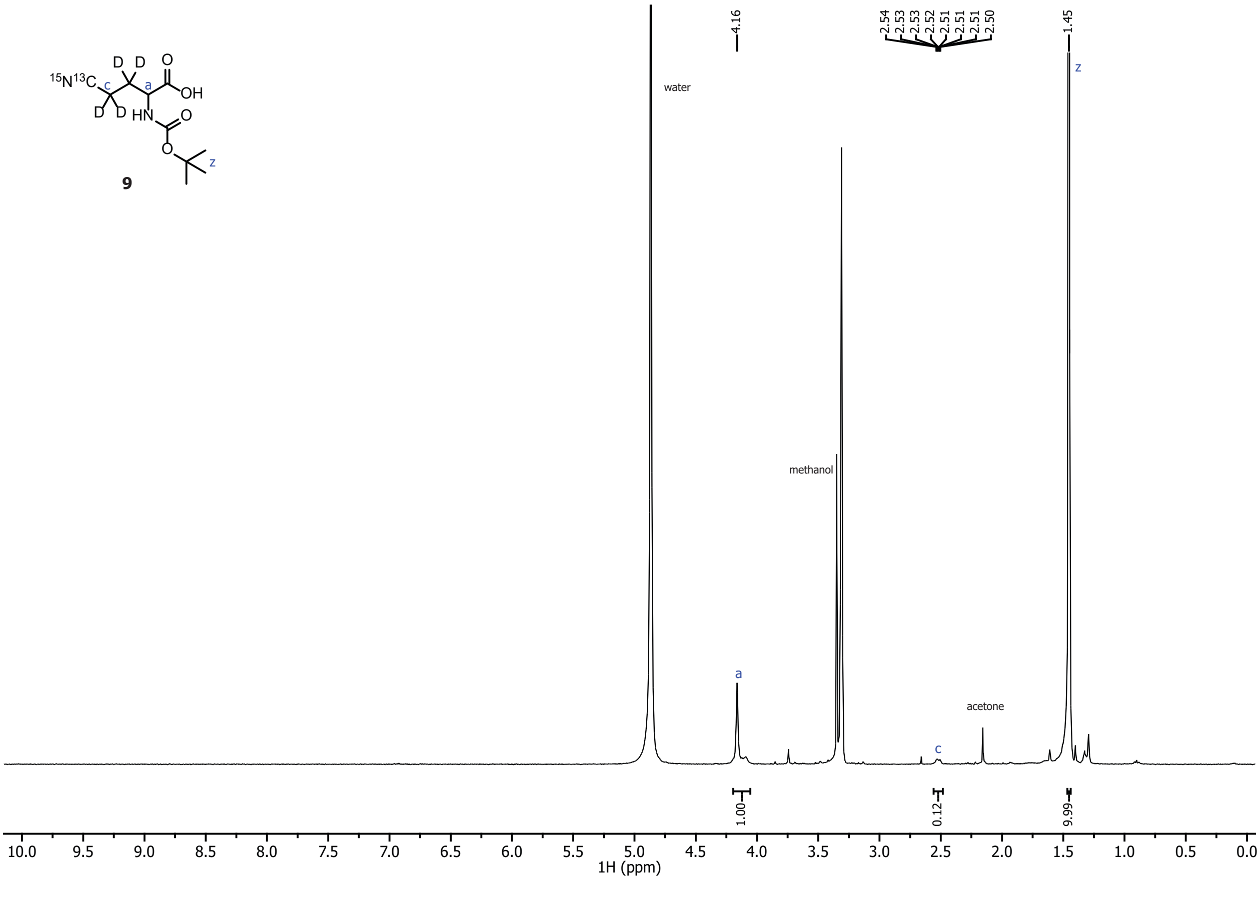

^{13}C NMR (101 MHz, MeOD)

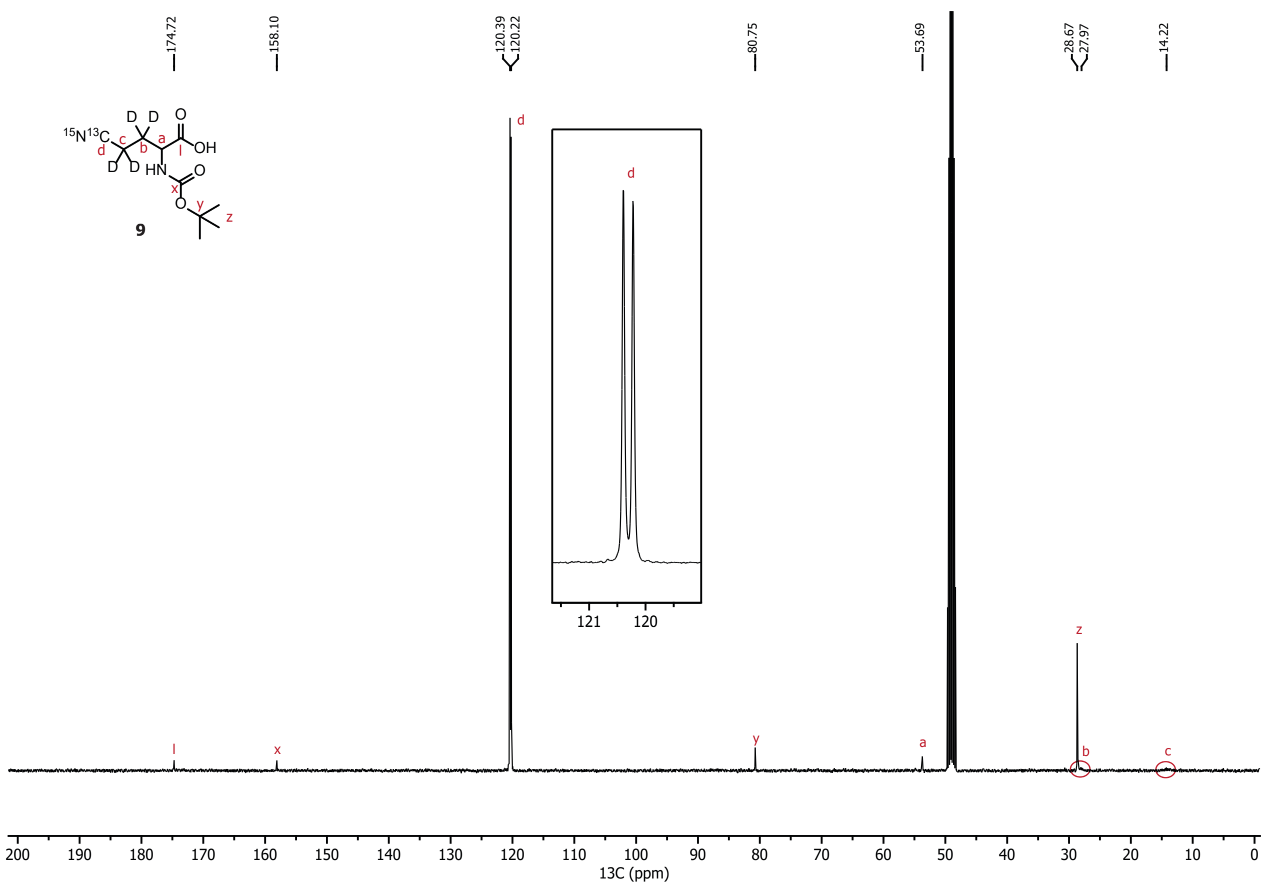

^{15}N NMR (61 MHz, MeOD)

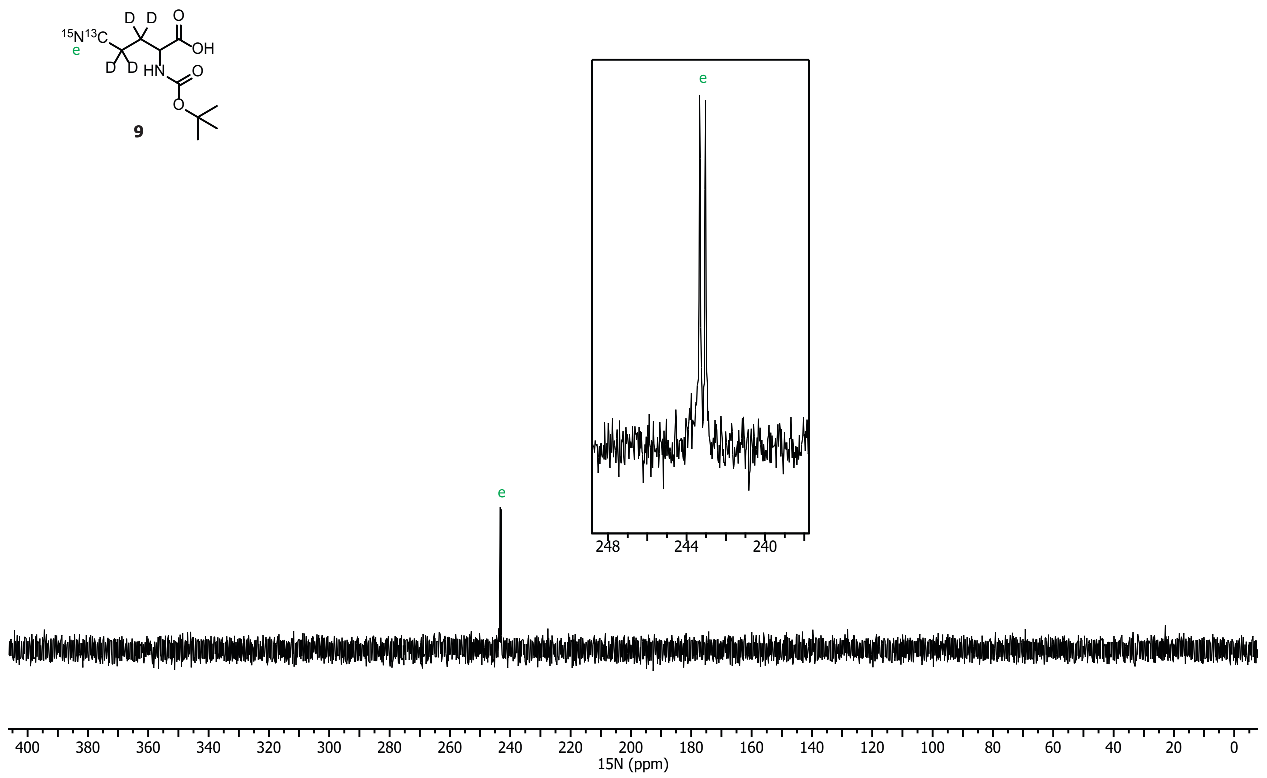

$^{13}C_{\delta}/^{2}H_{\beta,\gamma}/^{15}N_{\varepsilon}$ *N*-(*tert*-butoxycarbonyl)-L-ornithine (10)

^{1}H NMR (600 MHz, MeOD)

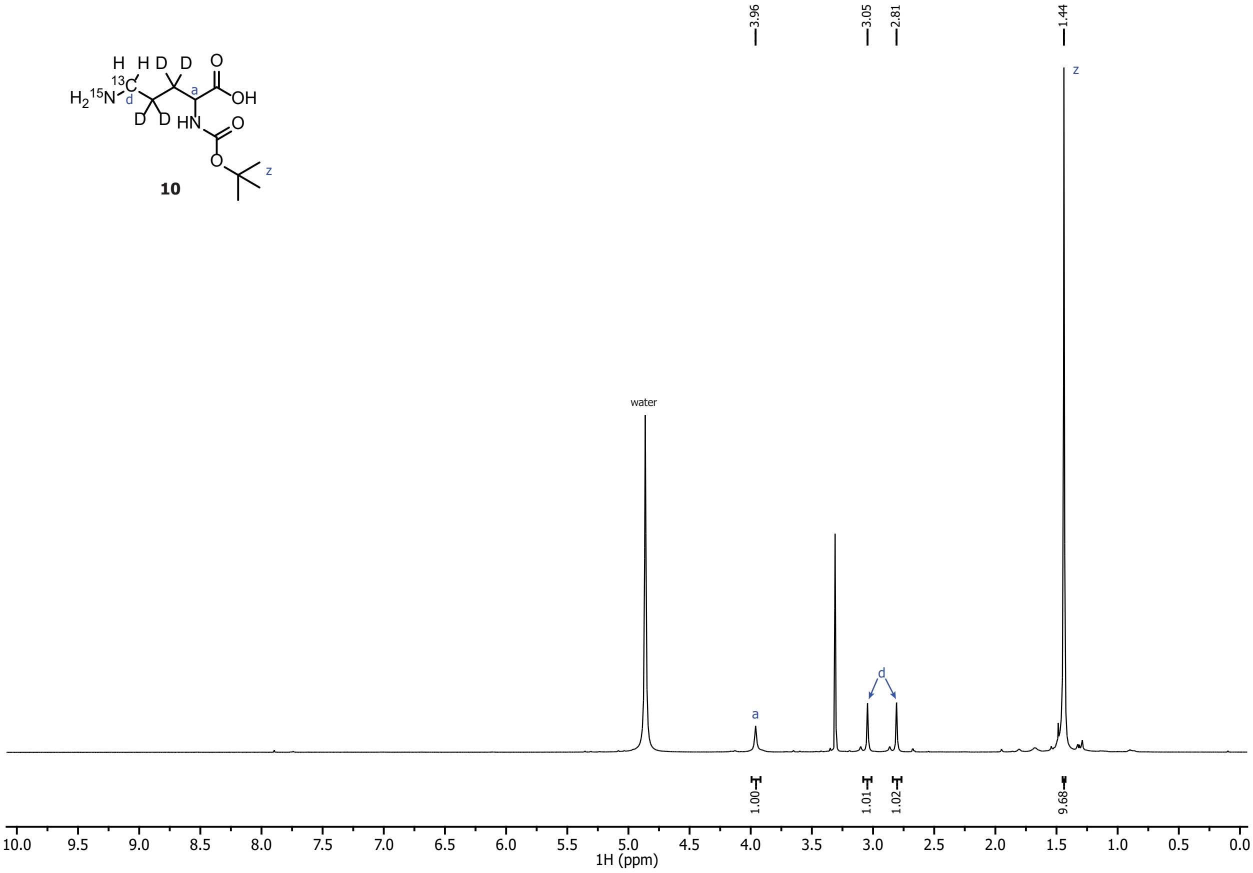

^{13}C NMR (101 MHz, MeOD)

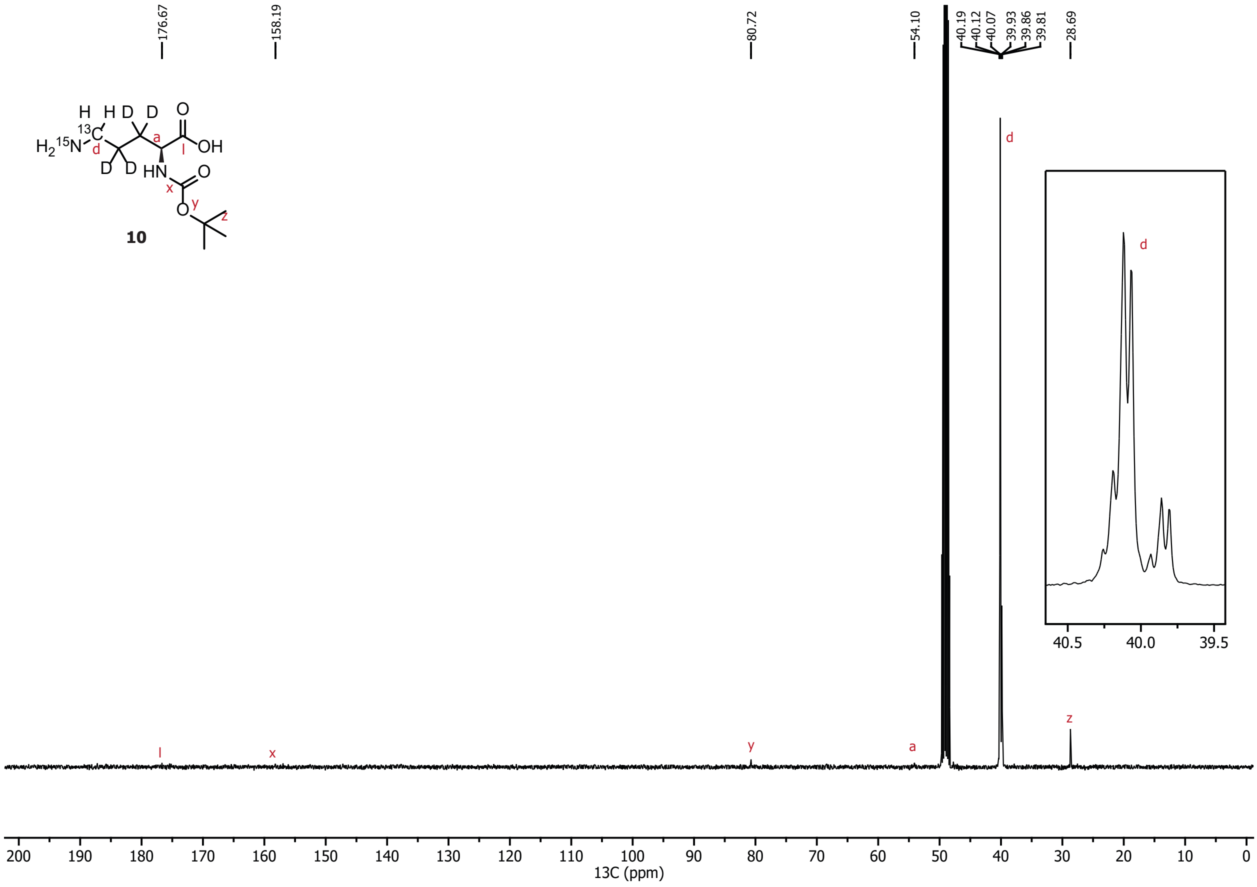

^{15}N NMR (61 MHz, MeOD)

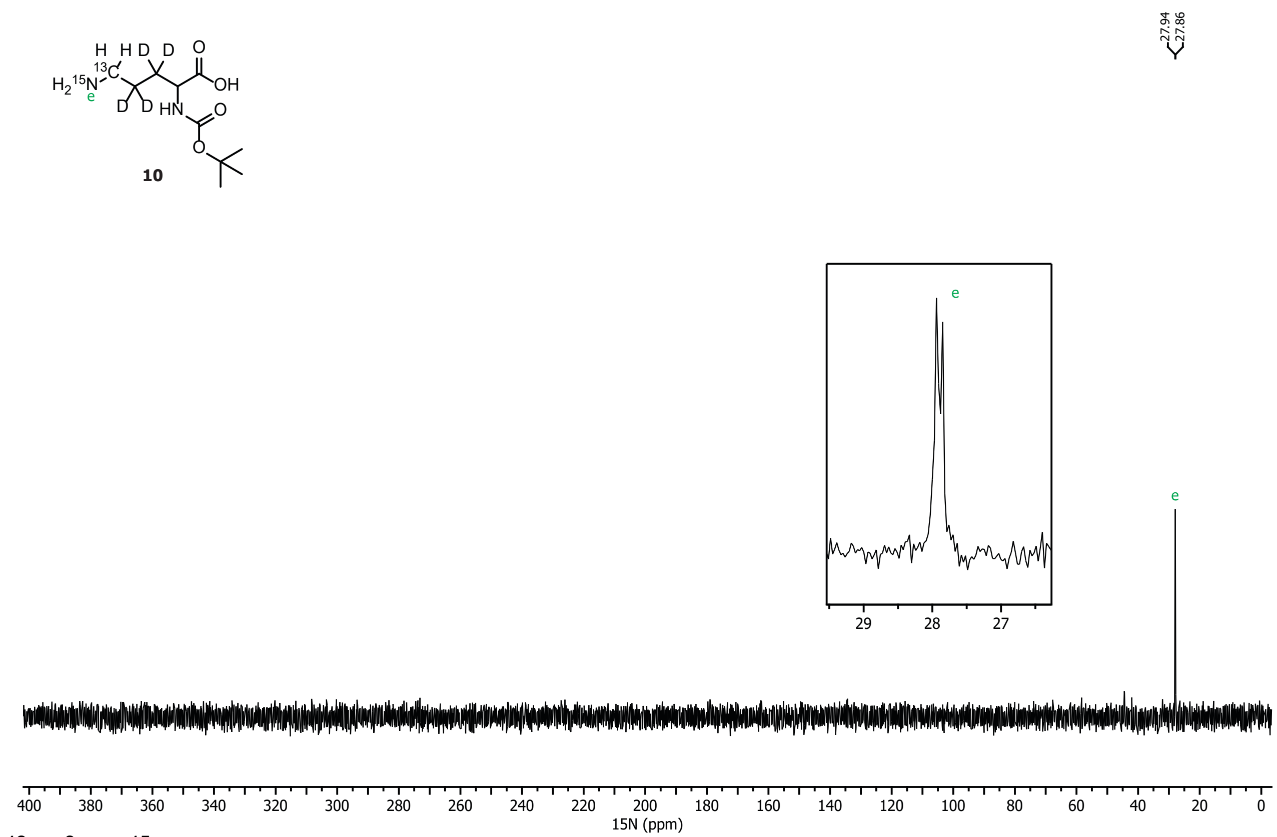

^{13}C$_{\delta}$/^{2}H$_{\beta,\gamma}$/^{15}N$_{\varepsilon}$ *N,N,N*-tris(*tert*-butoxycarbonyl)-L-arginine (11)

^{1}H NMR (400 MHz, CDCl$_3$)

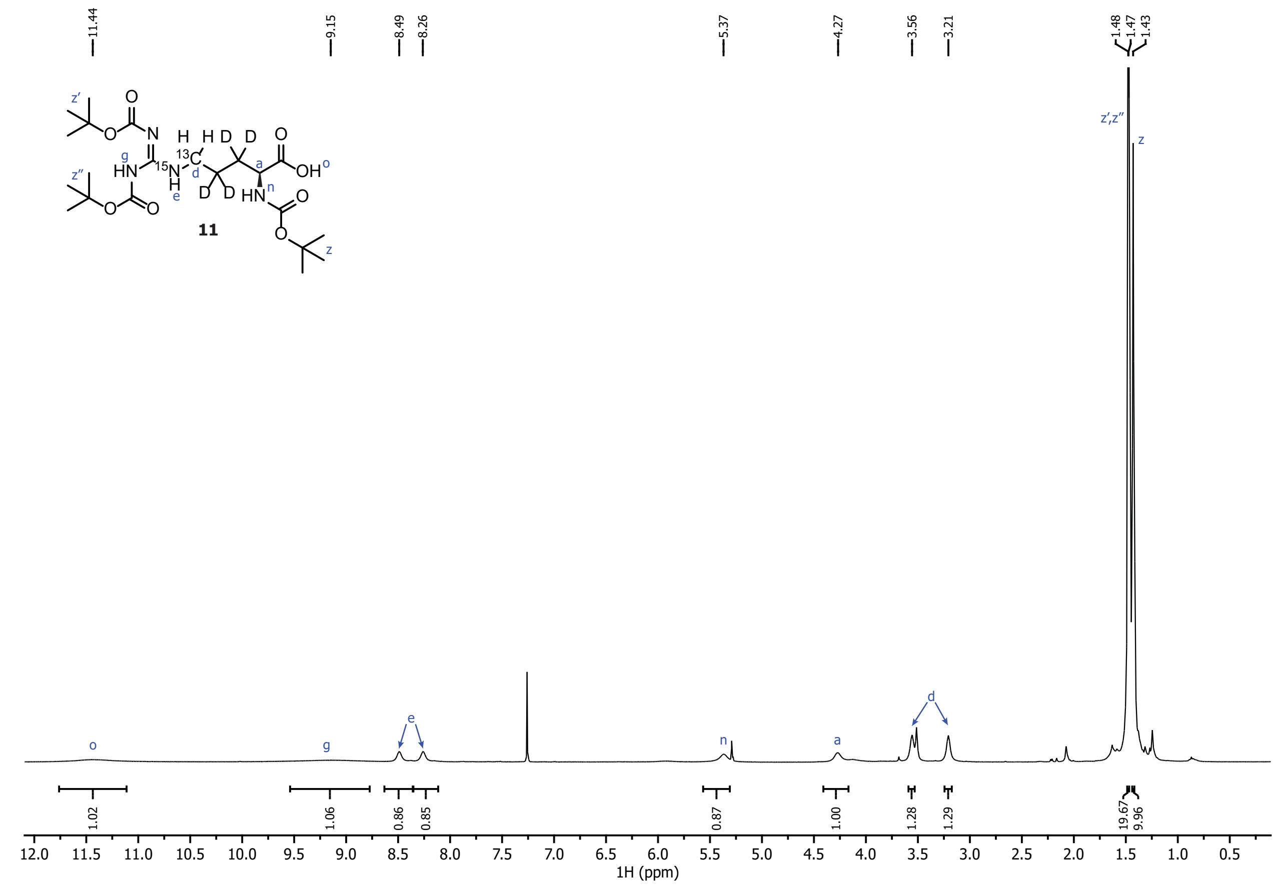

^{13}C NMR (151 MHz, $CDCl_3$)

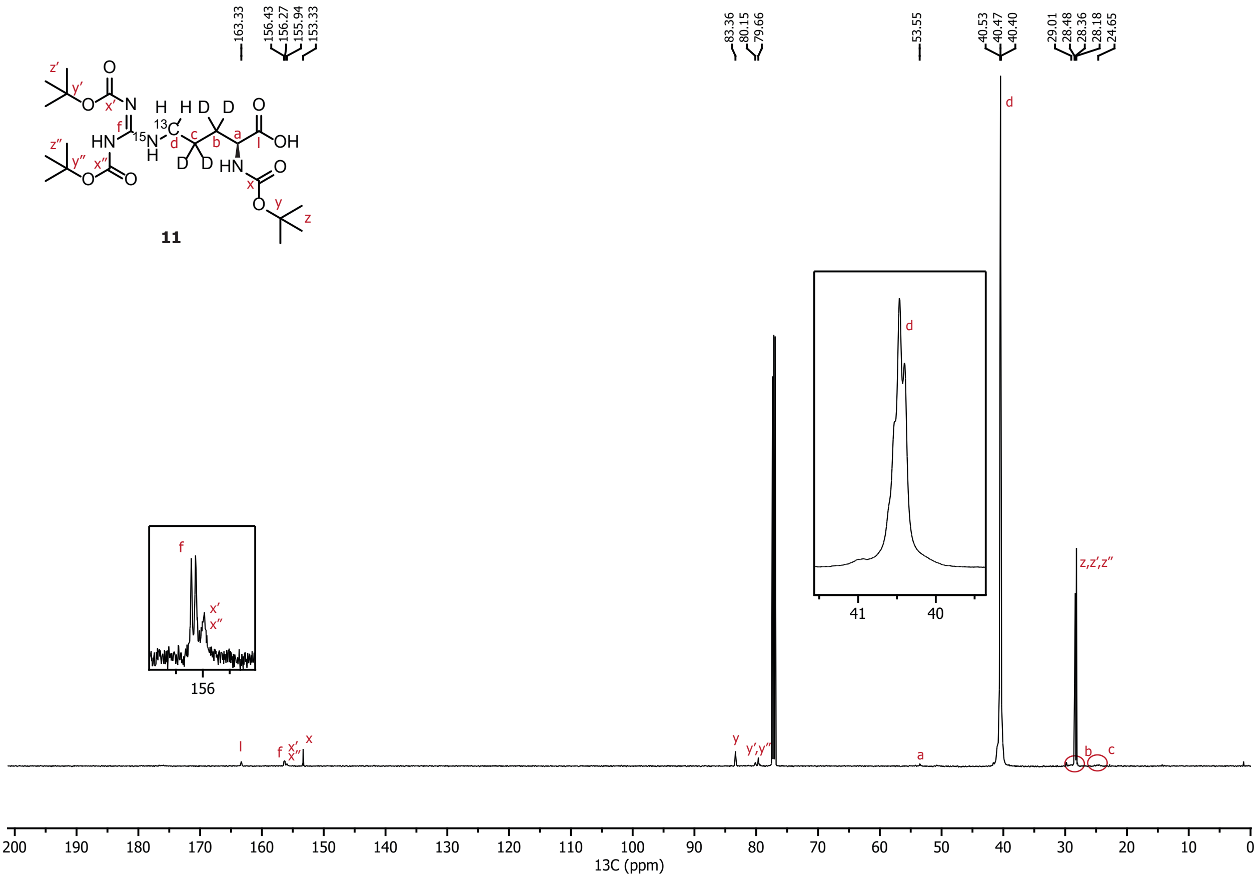

^{15}N NMR (61 MHz, $CDCl_3$)

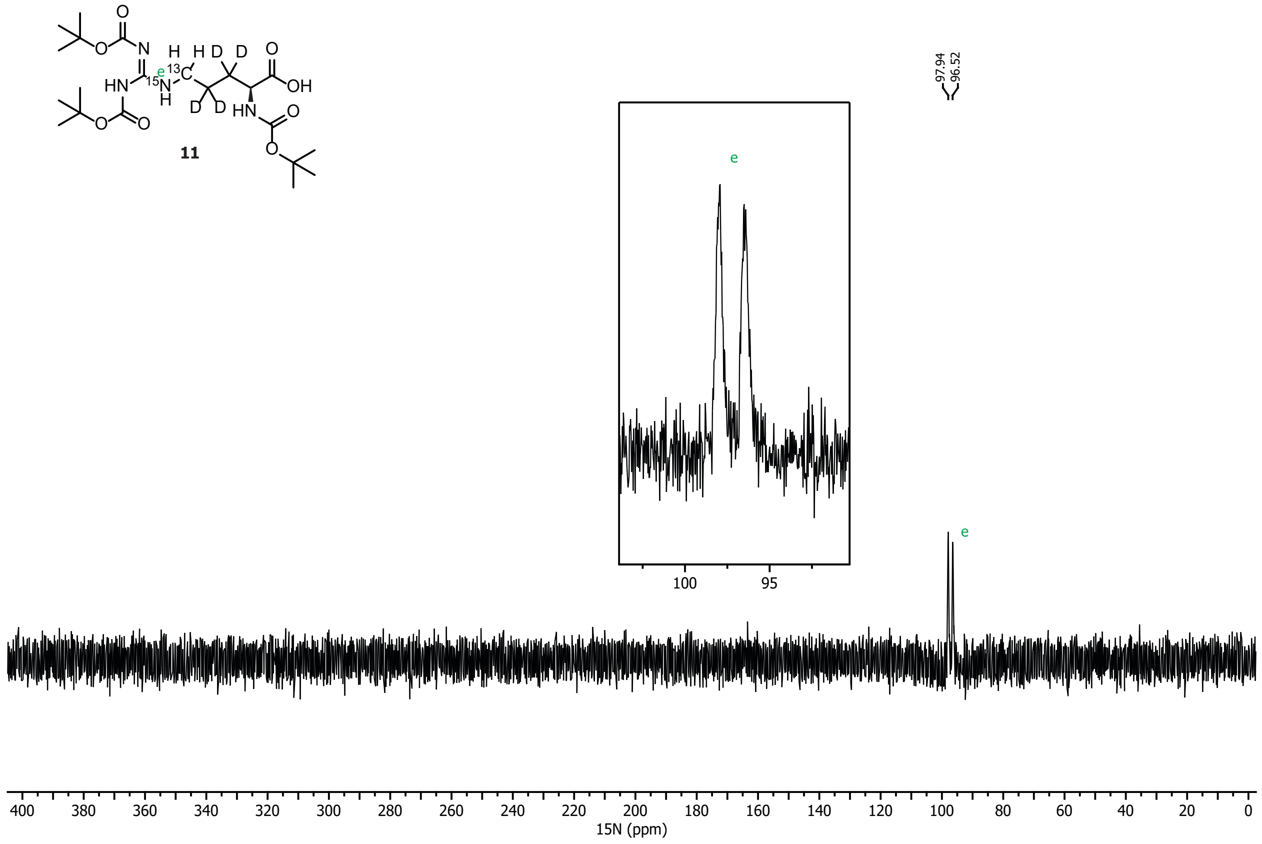

$^{13}C_{\delta}/^{2}H_{\beta,\gamma}/^{15}N_{\varepsilon}$ L-arginine (12)

^{1}H NMR (400 MHz, D_2O)

12

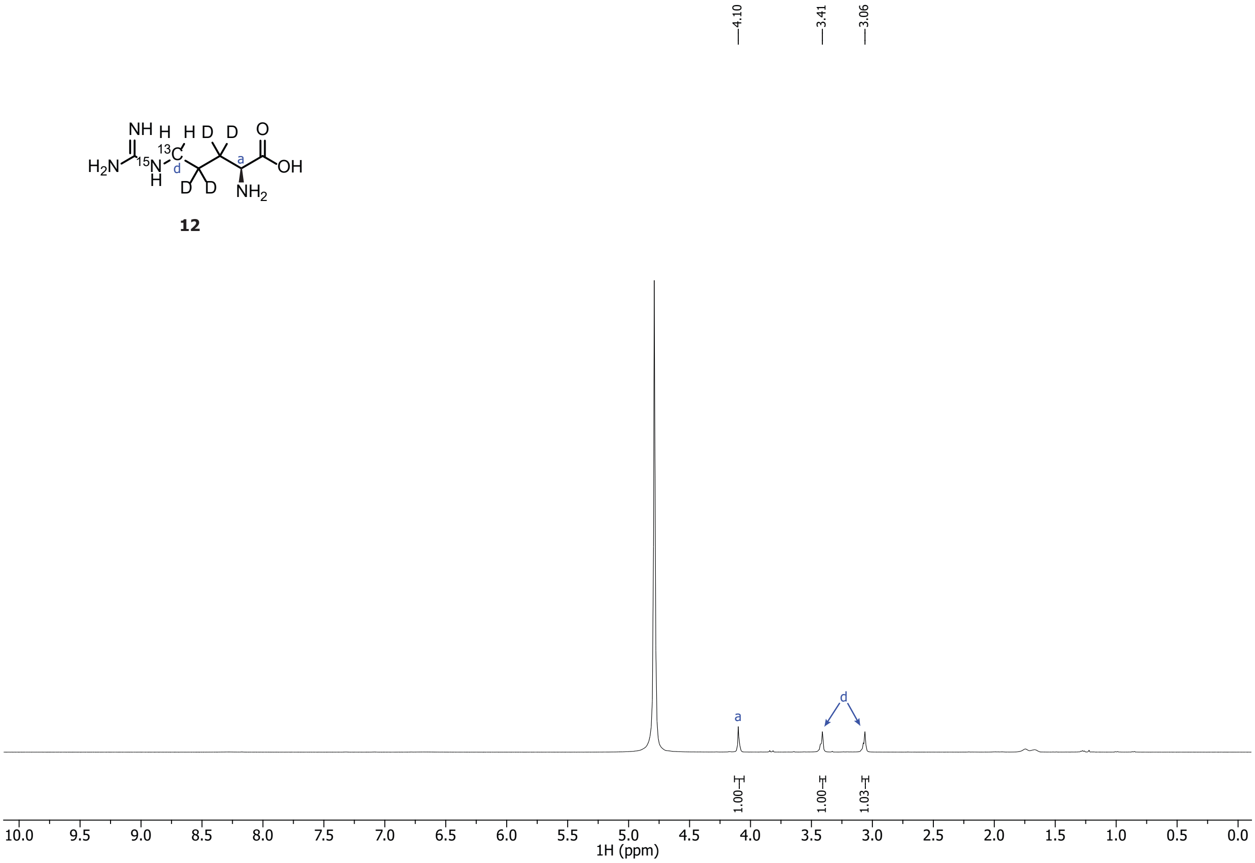

^{1}H NMR (600 MHz, DMSO-d_6)

12

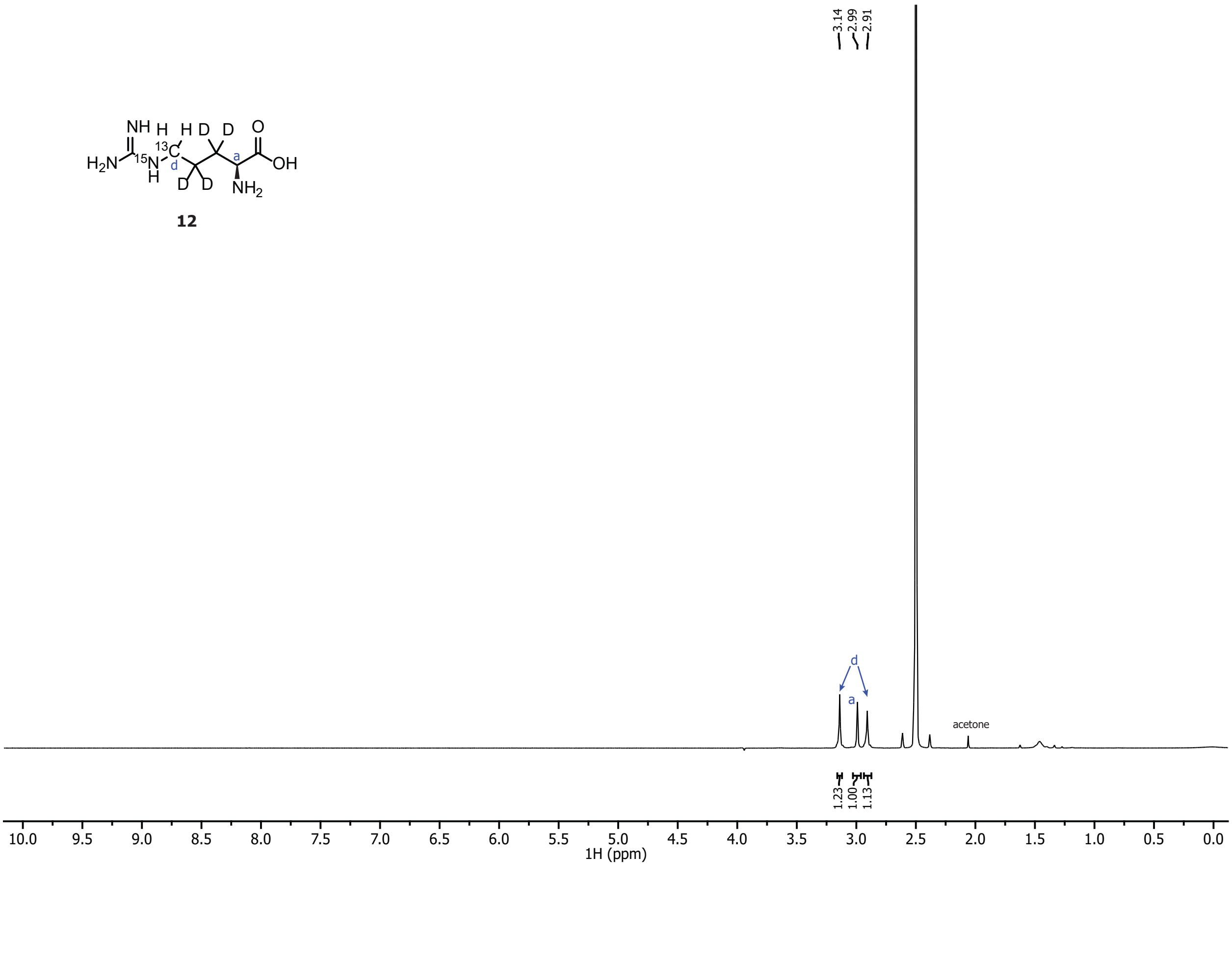

^{1}H NMR (600 MHz, MeOD)

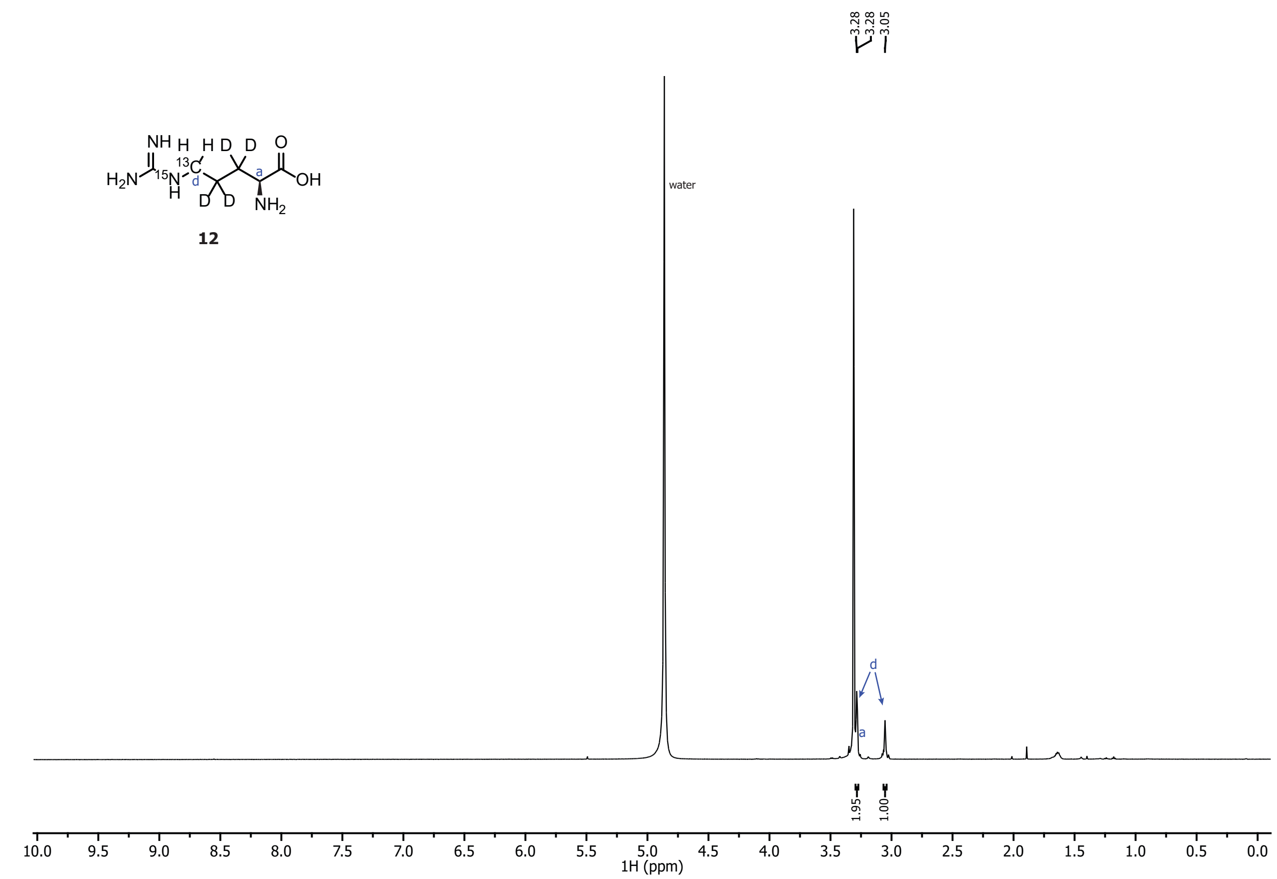

^{13}C NMR (151 MHz, D_2O)

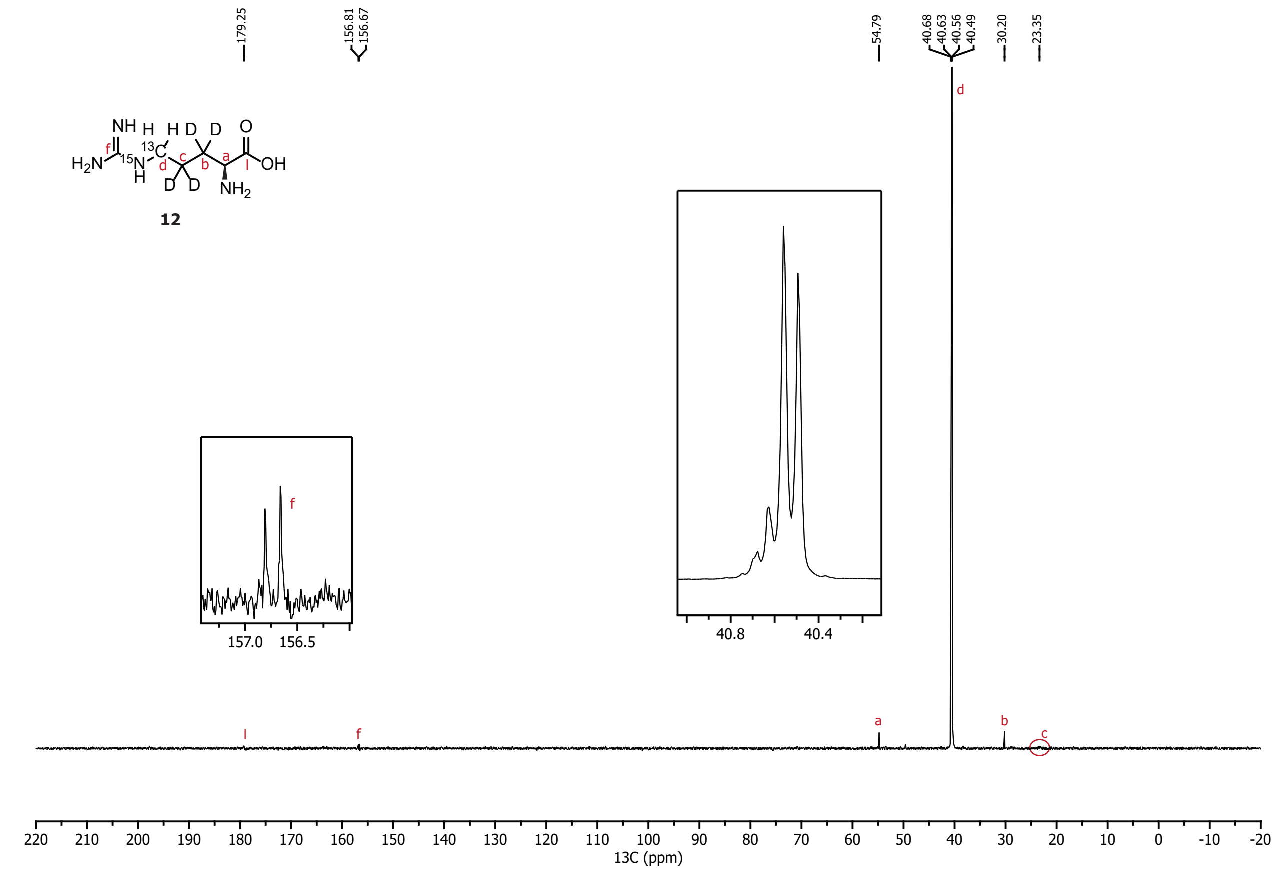

^{15}N NMR (61 MHz, D_2O)

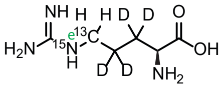

12

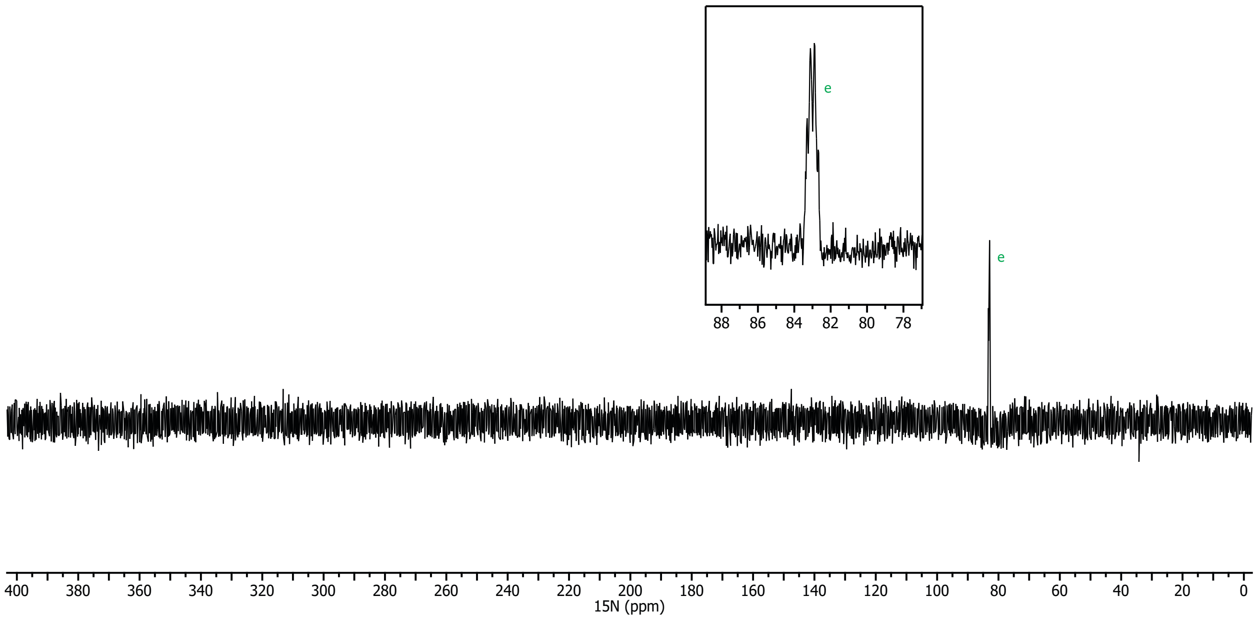

Supplement: Supplementary file 1 — Supporting Information [file CHEM-31-e202500408-s001.pdf]
